# Biotransformation of Tire-Derived 6PPD and 6PPD‑Q in Soil Nematode *Caenorhabditis elegans*: Unraveling Novel Phosphorylation Products and Distinct Kinetic Profiles

**DOI:** 10.1021/acs.est.5c02072

**Published:** 2025-07-08

**Authors:** Wei Wang, Gefei Huang, Fangfang Miao, Zhongying Zhao, Zongwei Cai

**Affiliations:** † State Key Laboratory of Environmental and Biological Analysis, Department of Chemistry, 26679 Hong Kong Baptist University, Hong Kong SAR 999077, China; ‡ Department of Biology, 26679 Hong Kong Baptist University, Hong Kong SAR 999077, China; ⊥ Eastern Institute of Technology, Ningbo 315200, China

**Keywords:** Suspect and nontargeted screening, phosphorylation, transformation products, tire-derived contaminants, soil ecosystem, *Caenorhabditis elegans*

## Abstract

The extensive use of tire antioxidant *N*-(1,3-dimethylbutyl)-*N*′-phenyl-*p*-phenylenediamine (6PPD) has raised significant environmental concerns, given the ubiquity and severe toxicity of 6PPD and its quinone derivative (6PPD-Q). While their hazards could be mediated through biotransformation pathways of detoxification and/or bioactivation and associated metabolites, the biotransformation of these tire-derived contaminants in soil organisms, key environmental compartments directly exposed to tire wear, remains unexplored. In this work, we investigated the biotransformation of 6PPD and 6PPD-Q in soil nematode *Caenorhabditis elegans* (*C. elegans*). We identified 9 transformation products (TPs) of 6PPD and 26 of 6PPD-Q using suspect and nontargeted screening methods, providing the first comprehensive metabolic profile of these contaminants in soil nematodes. Novel *in vivo* metabolites including phosphorylated and monohydroxy phosphorylated 6PPD-Q were first discovered, revealing unique metabolic pathways of these contaminants in *C. elegans* compared to other eukaryotes. Kinetic profiling delineates heterogeneous temporal patterns among TPs, with specific derivatives showing progressive accumulation over the exposure duration. Notably, isomers such as dihydroxy 6PPD-Q exhibit distinct substitution-site-dependent dynamics. Furthermore, *in silico* toxicity prediction indicated that certain TPs exhibited earthworm toxicity comparable to 6PPD-Q, implying potential contributions to the discernible adverse effects observed in *C. elegans*. Our findings elucidate the biotransformation of tire-derived contaminants in soil nematodes, suggest potential ecological concerns posed by 6PPD and 6PPD-Q to soil organisms, and underscore the necessity for further evaluation of their bioactivities and environmental impacts.

## Graphical abstract

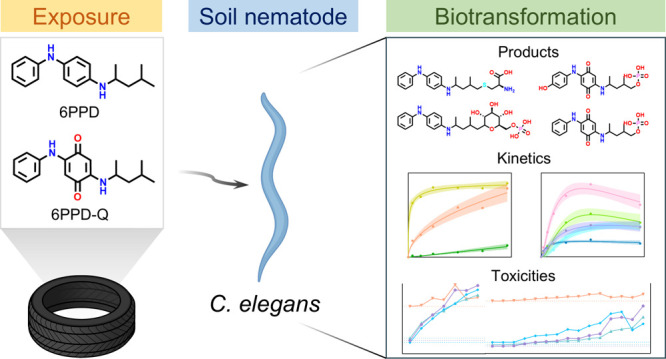

## Introduction

Biological transformation of commercial products can lead to highly toxic metabolites. For instance, oxybenzone is metabolized by sea anemones (*Aiptasia*) and mushroom corals (*Discosoma*) into phototoxic glucoside conjugates, leading to increased mortality of both organisms under UV exposure.^1^ Another example involves the cyanobacterium (*Aetokthonos hydrillicola*), which utilizes environmental bromide to synthesize neurotoxic metabolite of aetokthonotoxin, the causative agent of vacuolar myelinopathy, and is highly toxic to multiple species including nematode (*Caenorhabditis elegans*, LC_50_ 40 nM) and zebrafish (*Danio rerio*, LC_50_ 275 nM).^2^ These cases highlight the critical need to comprehensively elucidate the biotransformation of chemicals from commercial products, particularly the identity and toxicity of their transformation products (TPs) and associated metabolic pathways. *N*-(1,3-Dimethylbutyl)-*N*′-phenyl-*p*-phenylenediamine (6PPD) has been extensively employed as an antioxidant in rubber products, such as tires, footwear, synthetic turf infill, and playgrounds.^4^ Both 6PPD and its quinone derivative (6PPD-Q) have raised global environmental concerns due to their widespread occurrence across various environmental matrices, including water,^5^ soil,^6^ dust,^7,8^ and particulate matter,^58^ as well as their potent toxicity.^5^ 6PPD is classified as highly toxic to zebrafish, with a 96 h-LC_50_ of 2.2 mg/L.^9^ It induces severe eye damage and impairs embryonic growth and development, as evidenced by reduced hatchability, suppressed spontaneous movement, decreased body length, and increased deformity rates.^10^ As the culprit of urban runoff mortality syndrome, 6PPD-Q induces acute mortality in coho salmon (*Oncorhynchus kisutch*) at ultratrace levels (24 h-LC_50_ 95 ng/L)^11^ and causes similar acute fatalities in other salmonids, including brook trout (*Salvelinus fontinalis*, 24 h-LC_50_ 0.59 μg/L), rainbow trout (*Oncorhynchus mykiss*, 72 h-LC_50_ 1 μg/L),^12^ and white-spotted char (*Salvelinus leucomaenis pluvius*, 24 h-LC_50_ 0.51 μg/L).^13^ Additionally, 6PPD-Q has been found hazardous to other fish species, such as zebrafish larvae (24 h-LC_50_ 309 μg/L)^14^ and Chinese rare minnow (*Gobiocypris rarus*, 96 h-LC_50_ 162–201 ng/L).^15^ The species-specific toxicity of 6PPD-Q was mechanistically linked to tissue-specific impairment of mitochondrial respiration,^16^ and differential basal expression of biotransformation enzymes caused metabolic variations.^17^ Besides aquatic species, 6PPD and 6PPD-Q also exhibit significant toxicity to terrestrial organisms. Both contaminants bioaccumulate in the liver dose-dependently and induce hepatotoxicity in murine models.^18^ Our previous study demonstrated the rapid penetration of 6PPD-Q across the blood–brain barrier in mice within 0.5 h and revealed its metabolism and accumulation patterns by elucidating its TPs and toxicokinetics.^19^ Beyond mammals, 6PPD-Q is also toxic to other terrestrial species, particularly soil-dwelling organisms. Recent studies have revealed that 6PPD-Q disrupts multiple physiological processes in the nematode *Caenorhabditis elegans* (*C. elegans*), including lipid accumulation, neurotoxicity, mitochondrial dysfunction, intestinal damage, and impaired reproductive capacity, accompanied by reduced lifespan.^20–23^ These effects are mediated through perturbations in lipid metabolism, reproductive health, neuronal function, mitochondrial integrity, and gut physiology. The marked toxicity of 6PPD and 6PPD-Q to *C. elegans* suggestes a significant hazard to the soil ecosystem. However, their biotransformation characteristics in *C. elegans* remain uncharacterized, leaving critical knowledge gaps regarding their modes of action.

Soil, as the primary nutrient reservoir, is also the environmental compartment most directly exposed to rubber products, such as tires, cables, and artificial turf, due to their widespread use, placement, and disposal directly onto or into soil. Substantial amounts of 6PPD and 6PPD-Q can be released into the soil environment, posing potential ecological risks, as their presence in various soil samples, including roadside soil (6PPD: 31.4–831 ng·g^–1^; 6PPD-Q: 9.50–936 ng·g^–1^), industrial area soil (6PPD: 0.008–14.6 ng·g^–1^; 6PPD-Q: 0.002–4.4 ng·g^–1^), and green-belt soil (6PPD: 3–233 ng·g^–1^), has been well documented.^24–26^ Given the severe hazards of 6PPD and 6PPD-Q to *C. elegans* and their pervasive presence in soil environments, elucidating their biotransformation products and pathways, thus estimating their ecotoxicities, is imperative. In this study, we aim to (1) characterize the biotransformation of 6PPD and 6PPD-Q in *C. elegans*, thereby assessing their metabolic activities in soil organisms; (2) evaluate their transformation kinetics toe elucidate their bioavailability and bioaccumulation; and (3) assess the ecological toxicity of the identified TPs to estimate their broader ecological impacts. Our findings uncover novel biotransformation products of 6PPD and 6PPD-Q in a soil invertebrate model and demonstrate an integrated workflow that combines nontargeted metabolite screening with *in silico* toxicity prediction to prioritize bioactive derivatives for further toxicological characterization.

## Materials and Methods

### Chemicals and Cultures

The standard of 6PPD was purchased from TCI Chemical Co. (Shanghai, China) with purities higher than 98%. Potassium dihydrogen phosphate (KH_2_PO_4_), sodium phosphate dibasic (Na_2_HPO_4_), sodium chloride (NaCl), sodium hydroxide (NaOH), and magnesium sulfate (MgSO_4_) are GR grade that were purchased from Fisher Scientific (Hong Kong). The 6PPD-Q standard and the internal standard of deuterated 6PPD-Q (6PPD-Q-*d*_5_) were synthesized in the laboratory as reported previously.^24^ Surrogate standard diphenylamine-d_10_ was obtained from TRC (Burlington, Canada), while formic acid (FA, 99%) and other solvents such as dichloromethane (DCM) and methanol (MeOH) are HPLC grade and purchased from VMR chemicals (Hong Kong). Nematode growth medium (NGM) was made according to a previous protocol.^27^ M9 buffer, a liquid medium for *C. elegans* routine maintenance, was prepared by mixing 6.0 g of Na_2_HPO_4_, 3.0 g of KH_2_PO_4_, 5.0 g of NaCl, and 1 mL of 1 M MgSO_4_ in 1 L of distilled ultrapure water. Both NGM and M9 buffers were autoclaved for 120 min to remove any bacterial contamination before use. To obtain pure *Escherichia coli* (*E. coli*) OP50 lawns, we inoculated the fresh powder of OP50 with an empty NGM plate and picked up a single colony from the surface of the agar. The OP50 colony was then incubated in autoclaved Luria–Bertani broth (Sigma-Aldrich, St. Louis, US) at 37 °C for 12 h with continuous shaking, after which the overnight culture was spread onto the NGM agar.

### Preparation of *C. elegans* Strains

The wild-type Bristol N2 strain was collected from the Caenorhabditis Genetics Center (CGC), University of Minnesota, USA. A new stock of N2 worms was thawed and raised for five generations before use. To generate offspring and synchronize their stages, adult hermaphrodites were lysed by bleaching solution (0.5 M sodium hydroxide and 0.2 M hypochlorite), and the eggs were released into the autoclaved M9 buffer for a 16 h incubation. Hatched L1 larvae were raised on the surface of 90 mm NGM plates seeded with OP50 bacterial lawns for larval development. We collected L4 stage worms (i.e., about 38 h after feeding on NGM plates) for the exposure experiments.

### Exposure Experiments

Both 6PPD and 6PPD-Q powders were weighed and dissolved in organic carriers (i.e., methanol for 6PPD and DMSO for 6PPD-Q) to make 100 mg L^–1^ stock solutions. The exposure medium was prepared by diluting stock solutions 1000 times with autoclaved M9 buffer. About 10,000 worms were required to generate cellular pellets sufficient for the biotransformation analysis. To count the number of worms, the worm pellet was resuspended in 1 mL of M9 buffer and vortexed briefly. We transferred 5 μL of the solution to a 3% agarose pad and anesthetized nematodes with 1% levamisole. The number of worms was counted under a 30× magnification dissection microscope. Worms were transferred to clean 1.5 mL centrifuge tubes and rinsed with M9 buffer for five rounds to eliminate bacteria. Due to the rapid lifecycle and growth speed of *C. elegans*, acute exposure mode of 6PPD (24 h) and 6PPD-Q (48 h) was performed using L4 stage worms. A single-dose exposure was applied in M9 liquid medium (without *E. coli* OP50), with 6PPD and 6PPD-Q introduced at a concentration of 100 μg·L^–1^ in 10 mL of liquid medium. This exposure concentration was selected based on lethality assays (Figure S1) and detection limits of the TPs (Text S1). *E. coli* OP50 was excluded to avoid bacterial metabolism of 6PPD and 6PPD-Q or interactions with the test compounds.^28^ For 6PPD (half-life ≈ 8 h), the medium was replenished once at 12 h to compensate for degradation observed in external concentrations (Figure S2), while 6PPD-Q (half-life > 33 h) did not require replenishment.^29^ This exposure method mimics episodic tire-derived chemicals in natural environments^30^ and has been widely adopted for biotransformation of 6PPD-Q in other species.^31–33^ All nematodes were maintained in 12-well plates on a shaker at 100 rpm to prevent sedimentation. For 6PPD exposure, samples were collected at eight time points: 0, 0.5, 1, 3, 6, 12, 18, and 24 h, for kinetic analysis. For 6PPD-Q, seven time points were used: 0, 1, 3, 6, 12, 24, and 48 h. Three biological replicates were performed for each time interval.

### Collection of Supernatant and Worm Extracts

Worms were centrifuged at 3000*g* for 1 min to separate worm pellets from the liquid medium (denoted as the supernatant) at the end of the exposure period. The resulting supernatant was filtered through a 0.22 μm nylon filter, spiked with 20 ng of surrogate standard, and further extracted using Waters HLB by a solid phase extraction (SPE) cartridge. Generally, the cartridge was activated by using DCM, MeOH, and water (3 mL each). After the sample was loaded, the cartridge was drenched with 5 mL of water, vacuum-dried for 15 min, and eluted with 3 mL of MeOH and 3 mL of DCM. The elutes were merged, blown to dryness, redissolved in 50 μL of MeOH, and added with 20 ng of internal standards before instrumental analysis. The worm pellet was washed extensively by an autoclaved M9 buffer for 5 times and resuspended in 1 mL of 80% MeOH. After spiking with 20 ng of the surrogate standard, nematodes were lysed by bead beating with 0.5 and 0.2 mm zirconium oxide beads at 0 °C for 3 min (20 s pulse and 10 s pause). Insoluble particles and beads were pelleted by ultracentrifugation at 16000*g* and 4 °C for 30 min. The resulting supernatant was transferred to a sterile 1.5 mL centrifuge tube, evaporated to dryness using a freeze-dry vacuum concentrator, resuspended in 50 μL of MeOH, and added with 20 ng of internal standards for further analysis.

### Suspect and Nontargeted Screening Analysis

The screening and identification of biotransformation TPs were conducted using ultraperformance liquid chromatography (UPLC, Vanquish MD) integrated with a high-resolution hybrid quadrupole-Orbitrap mass spectrometer (Q Exactive, Thermo Scientific, USA) operated in both positive and negative electron spray ionization (ESI) modes. Data-dependent MS^2^ (ddMS^2^) acquisition was utilized to obtain the full-scan molecular-ion data and the MS^2^ fragments for nontargeted screening, while parallel reaction monitoring (PRM) with time-specific isolation windows and varied collision energies was employed to obtain the detailed structural information on the suspects. All instrumental parameters are detailed in Table S1. Newly formed products were defined as signals that met the criteria (i) abundance ≥ 10-fold above background levels and (ii) absence in blank controls.^34^ Metabolite identification and structural elucidation utilized HRMS data at a resolution of 140 000, with mass error thresholds <10 ppm between predicted and observed mass-to-charge ratio (*m*/*z*). An in-house candidate list of biotransformation products, which integrates known xenobiotic pathways in other organisms and predictive libraries based on functional-group alteration patterns, was compiled for suspect screening (Table S3). Nontargeted analysis combined background subtraction, isotopic pattern matching, and MS^2^ fragment verification. Details about the predictive library construction and nontargeted screening can be found in Text S2.

### Quantitative Analysis of Transformation Kinetics

To monitor the kinetics of biotransformation, the levels of 6PPD, 6PPD-Q, and their TPs were (semi-) quantified leveraging UPLC-tandem mass spectrometer (MS/MS) as our earlier study described.^24^ Specifically, a Vanquish MD UPLC system was coupled with an ESI triple quadrupole mass spectrometer (TSQ Altis, Thermo Scientific, USA) for targeted quantification of 6PPD, 6PPD-Q, and the identified TPs.

In the absence of commercial standards, TPs were semiquantified by multiple reaction monitoring (MRM) with optimized precursor–product ion transitions. Detailed method parameters, including collision energies and retention windows for each analyte, are listed in Table S2. The measured levels/abundances of 6PPD, 6PPD-Q, and their TPs were corrected using the ratio of the number of nematodes at a given time (*N*_*t*_) to the initial number (*N*_0_), to eliminate bias due to population size variation (Figure S1).^35^ An internal calibration method, with an internal standard of 6PPD-Q-*d*_5_, was adopted using eight-level calibration curves ranging from 0.01 to 100 μg·L^–1^, with all regression coefficients exceeding 0.99 (Figure S3). Samples were diluted if their levels were beyond the calibration ranges. Matrix effects (MEs) were evaluated by spiking 6PPD and 6PPD-Q into the extracts and cultures, respectively, and calculated as the ratio of the measured abundances in the matrix to the abundances of the analytes in pure solvent (Text S3). Three concentration levels (10, 50, and 100 μg·L^–1^) were tested in triplicate. The resulting ME for 6PPD was 101 ± 9% in cultures and 106 ± 13% in extracts, while for 6PPD-Q it was 102 ± 4% in cultures and 90 ± 5% in extracts.

### QA/QC, Data Processing, and Visualization

All glassware and quartz dishes were prerinsed with Milli-Q water and methanol to remove hydrophilic impurities and organic residues and then sterilized before use. Experiments were performed in triplicate, and the results were reported as the mean ± standard deviation (SD). Prior to MS analysis of each batch, blank quality-control samples (pure water and methanol) were processed and analyzed using the same method to identify potential system contamination and minimize cosolvent effects.^36^ The MS system was calibrated using manufacturer-supplied solutions to ensure a mass accuracy within 10 ppm. Surrogate standard recoveries in both the supernatant and worm pellet ranged from 70 to 130%.

Data analysis included MS data processing and *in silico* ecotoxicity estimation. MS data were processed by using Xcalibur software (v4.3.7, Thermo Scientific, USA), and statistical analyses were performed in SPSS (v11.0, IBM, USA). Structure elucidation of unknown TPs was supported by Mass Frontier software (version 8.0, Thermo Scientific, USA). The individual ecotoxicity of 6PPD, 6PPD-Q, and their TPs on representative aquatic and terrestrial species (fish, daphnia, green algae, and earthworms) was computationally predicted using the Ecological Structure–Activity Relationship (ECOSAR) model (v2.2) released by US EPA (Table S4).^37^ This validated algorithm estimates toxicity end points through structural analogies to reference compounds and has been endorsed by internationally recognized regulatory agencies of the US EPA, OECD, and EU.^38^ The model-derived acute toxicity parameters including 96 h median lethal concentration for fish (96 h fish LC_50_), 48 h LC_50_ for *Daphnia magna* (48 h daphnid LC_50_), 96 h median effective concentration for green algae (96 h green algae EC_50_), and 14 day LC_50_ for earthworms (14 d earthworm LC_50_), served exclusively for comparative toxicity ranking via QSAR-based prioritization but did not represent their absolute toxicological benchmarks. The visualization of the time-dependent kinetic curve of each analyte was subsequently calculated by fitting the dose–response curve with a best-fit model^39^ implemented on GraphPad Prism (GraphPad Software Inc., California, USA).

## Results and Discussion

### Identification of Biotransformation Products of 6PPD and 6PPD-Q

First, we combined suspect and nontargeted screening strategies to identify the biotransformation products of 6PPD and 6PPD-Q in *C. elegans*. Table 1 summarizes molecular information on the identified TPs: nine 6PPD metabolites and 26 6PPD-Q metabolites. These molecules were assigned varying Schymanski confidence levels^40^ based on their MS^2^ fragmentation annotations and structure elucidation. A variety of isomers sharing the same *m*/*z* but differing in retention times (RTs) were identified, likely due to position or functional group isomerism. Figure 1A depicts the RTs of all identified TPs, revealing a large range of more hydrophilic metabolites with shorter RTs for both 6PPD and 6PPD-Q. This pattern aligns with the established paradigm of xenobiotic metabolism, in which biotransformation pathways predominantly increase aqueous solubility via Phase II conjugation reactions (e.g., glucuronidation, sulfation), thereby facilitating renal or biliary elimination of hydrophobic compounds.^41^ Structural elucidation of representative TPs for 6PPD and 6PPD-Q, with typical conjugation reaction features, is shown in Figure 1B–E, while detailed interpretations of other TPs are provided in Figures S4–S11. The elucidation of 6DTP511 provides a representative example: its mass difference (Δ*m*/*z* 242.0174) relative to the parent compound 6PPD corresponds to the elemental composition C_9_H_11_O_8_P (mass error −7.4 ppm), consistent with a phosphoglucosidation transformation. To confirm this assignment, MS/MS fragmentation spectra of 6DTP511 were acquired in both positive (Figure 1B) and negative (Figure 1C) modes. In positive mode, characteristic fragments arising from the neutral loss of phosphoric acid (H_3_PO_4_) yielded the ion C_24_H_33_N_2_O_4_^+^ (mass error −2.4 ppm). Additional fragments at *m*/*z* 395.2321 and *m*/*z* 268.1939 imply the cleavage of a hexose unit, with sequential losses of H_2_O (indicative of a hydroxyl group) and C_6_H_11_O_3_ (may correspond to 1,3-dihydroxy-4-methyl-tetrahydrofuran). While in negative mode, diagnostic ions corresponding to dihydrogen phosphate (H_2_PO_4_^–^) and phosphite (PO_3_^–^) further support the identification of 6DTP511 as a phosphoglucoside conjugate of 6PPD. Previous studies in *C. elegans* indicate that glucoside phosphate conjugation preferentially occurs on amino groups when no free hydroxyl groups are present.^42,43^ This preference may explain the isomerization of 6DTP511, which arises from conjugation at different secondary amino sites on 6PPD. Similarly, we identified a phosphorylated monohydroxy 6PPD-Q metabolite, designated 6QTP411, which exhibited sequential losses of hydroxyl, dihydrogen phosphate, isobutyl, and ethyl groups in +ESI MS/MS (Figure 1D). Detection of diagnostic H_2_PO_4_^–^ (*m*/*z* 96.9696) and PO_3_^–^ (*m*/*z* 78.9591) ions in −ESI (Figure 1E) further confirms the phosphorylation of monohydroxy 6PPD-Q at the quinone moiety.

**Table 1:** Molecular Characteristics of the Identified Biological Transformation Products of 6PPD (Prefix 6DTP) and 6PPD-Q (Prefix 6QTP)

**TP name**	**Proposed transformation**	**Formula**	**Exact mass**	*m*/*z***(+)**	*m*/*z***(−)**	**RT (min)**	**Confidence level**	**MS** ^**2**^ **fragments**
6DTP388	6PPD-cysteinyl	C_21_H_29_N_3_O_2_S	387.1980	388.2046	386.1908	9.07	Level 3	Figure S4
6DTP431	6PPD-N-glucoside	C_24_H_34_N_2_O_5_	430.2468	431.2540	429.2395	8.56	Level 3	Figure S4
6DTP511L	6PPD-N-glucoside-6-phosphate	C_24_H_35_N_2_O_8_P	510.2131	511.2204	509.2058	8.44	Level 3	Figure 1
6DTP511R	6PPD-N-glucoside-6-phosphate	C_24_H_35_N_2_O_8_P	510.2131	511.2204	509.2058	10.64	Level 3	Figure S4
6DTP186	4-HDPA	C_12_H_11_NO	185.0841	186.0913	184.0768	11.04	Level 2	Figure S4
6DTP348L	4-HDPA-N-glucoside	C_18_H_21_NO_6_	347.1369	348.1441	346.1296	7.89	Level 3	Figure S5
6DTP348R	4-HDPA-N-glucoside	C_18_H_21_NO_6_	347.1369	348.1441	346.1296	10.04	Level 3	Figure S5
6DTP428L	4-HDPA-N-glucoside-6-phosphate	C_18_H_22_NO_9_P	427.1032	428.1105	426.0959	7.73	Level 3	Figure S5
6DTP428R	4-HDPA-N-glucoside-6-phosphate	C_18_H_22_NO_9_P	427.1032	428.1105	426.0959	9.90	Level 3	Figure S6
6QTP297	Dehydrogenated 6PPD-Q	C_18_H_20_N_2_O	296.1525	297.1598	295.1452	9.66	Level 3	Figure S7
6QTP313	Dehydrogenated monohydroxy 6PPD-Q	C_18_H_20_N_2_O_3_	312.1474	313.1547	311.1401	8.41	Level 3	Figure S7
6QTP315L	Monohydroxy 6PPD-Q	C_18_H_22_N_2_O_3_	314.1630	315.1703	313.1558	11.57	Level 3	Figure S7
6QTP315M	Monohydroxy 6PPD-Q	C_18_H_22_N_2_O_3_	314.1630	315.1703	313.1558	13.12	Level 3	Figure S7
6QTP315R	Monohydroxy 6PPD-Q	C_18_H_22_N_2_O_3_	314.1630	315.1703	313.1558	14.09	Level 3	N.A.
6QTP329AL	Monohydroxy 6PPD-Q aldehyde	C_18_H_20_N_2_O_4_	328.1423	329.1496	327.1350	11.42	Level 3	Figure S8
6QTP329AR	Monohydroxy 6PPD-Q aldehyde	C_18_H_20_N_2_O_4_	328.1423	329.1496	327.1350	11.56	Level 3	Figure S8
6QTP331L	Dihydroxy 6PPD-Q	C_18_H_22_N_2_O_4_	330.1580	331.1652	329.1507	9.51	Level 3	Figure S8
6QTP331R	Dihydroxy 6PPD-Q	C_18_H_22_N_2_O_4_	330.1580	331.1652	329.1507	9.88	Level 3	Figure S8
6QTP349	Trihydroxy 6PPD hydroquinone	C_18_H_24_N_2_O_5_	348.1685	349.1758	347.1612	8.74	Level 3	Figure S9
6QTP477L	6PPD-Q O-glucoside	C_24_H_32_N_2_O	476.2159	477.2231	475.2086	9.78	Level 3	Figure S9
6QTP477R	6PPD-Q O-glucoside	C_24_H_32_N_2_O	476.2159	477.2231	475.2086	10.71	Level 3	Figure S9
6QTP493	Monohydroxy 6PPD-Q O-glucoside	C_24_H_32_N_2_O_9_	492.2108	493.2181	491.2035	7.83	Level 3	Figure S9
6QTP395L	6PPD-Q phosphoryl	C_18_H_23_N_2_O_6_P	394.1294	395.1366	393.1221	9.53	Level 3	Figure S10
6QTP395R	6PPD-Q phosphoryl	C_18_H_23_N_2_O_6_P	394.1294	395.1366	393.1221	9.67	Level 3	N.A.
6QTP411	Monohydroxy 6PPD-Q phosphoryl	C_18_H_23_N_2_O_7_P	410.1243	411.1316	409.1170	8.58	Level 3	Figure 1
6QTP317	Monohydroxy 6PPD hydroquinone	C_18_H_24_N_2_O_3_	316.1787	317.1860	315.1714	11.85	Level 3	Figure S10
6QTP361L	Methoxy dihydroxy 6PPD-Q	C_19_H_24_N_2_O	360.1685	361.1779	359.1612	9.27	Level 3	N.A.
6QTP361M	Methoxy dihydroxy 6PPD-Q	C_19_H_24_N_2_O	360.1685	361.1779	359.1612	10.53	Level 3	N.A.
6QTP361R	Methoxy dihydroxy 6PPD-Q	C_19_H_24_N_2_O	360.1685	361.1779	359.1612	10.65	Level 3	Figure S10
6QTP218	2-hydroxyl-5-phenylamino-1,4-quinone	C_12_H_11_NO	217.0739	218.0812	216.0666	4.85	Level 4	N.A.
6QTP329L	Methoxy 6PPD-Q	C_19_H_24_N_2_O_3_	328.1787	329.1860	327.1717	15.89	Level 3	Figure S10
6QTP329R	Methoxy 6PPD-Q	C_19_H_24_N_2_O_3_	328.1787	329.1860	327.1714	16.80	Level 3	N.A.
6QTP347	Trihydroxy 6PPD-Q	C_18_H_22_N_2_O_5_	346.1529	347.1601	345.1456	8.08	Level 4	N.A.
6QTP345L	Methoxy monohydroxy 6PPD-Q	C_19_H_24_N_2_O	344.1736	345.1809	343.1663	9.64	Level 4	N.A.
6QTP345R	Methoxy 6PPD hydroquinone aldehyde	C_19_H_24_N_2_O	344.1736	345.1809	343.1663	12.01	Level 3	Figure S11

**Figure 1:**
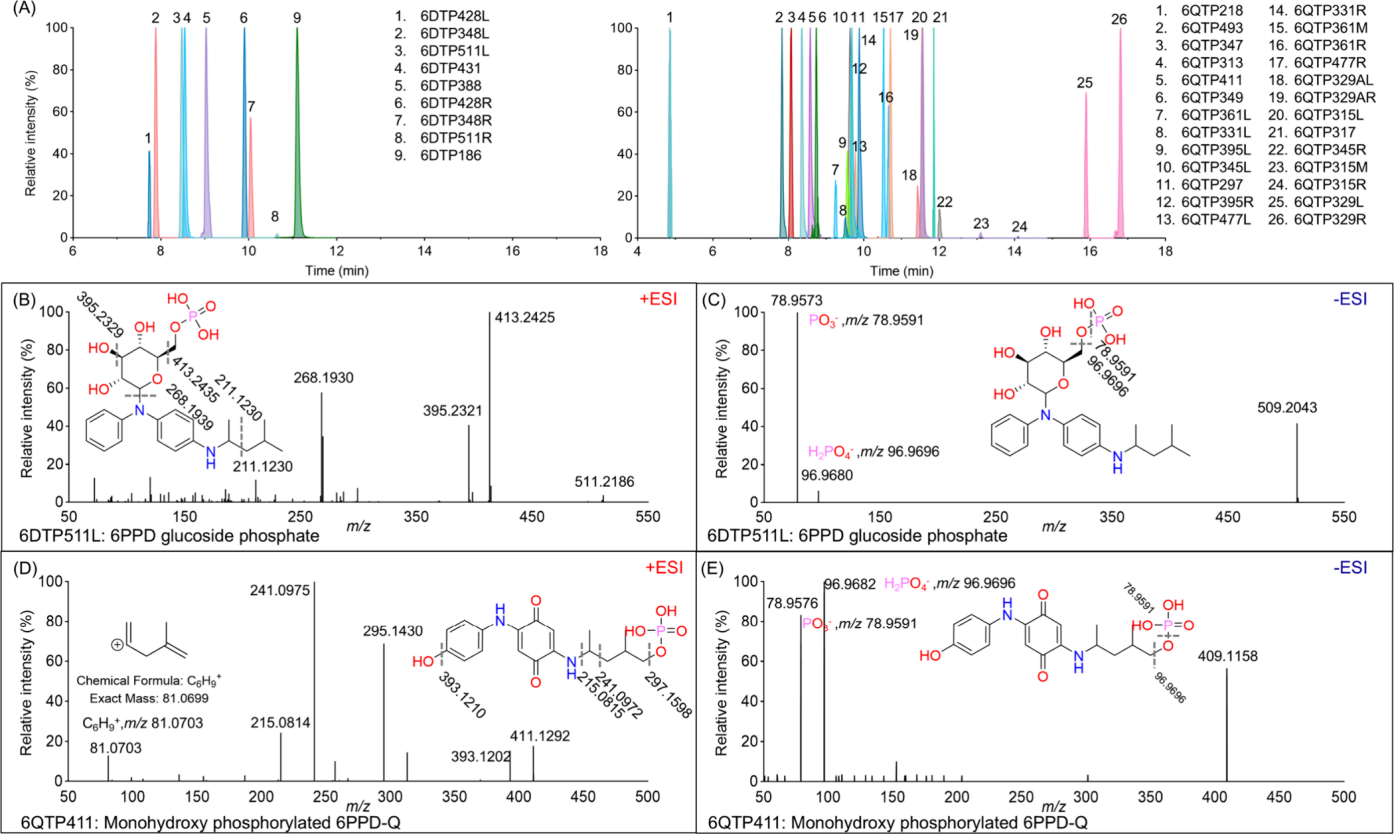
(A) Extracted ion chromatographs for biotransformation products of 6PPD and 6PPD-Q from exposure to *C. elegans*. Chromatographic peak intensities were normalized by their respective base peaks. Isomers are labeled with the same color. MS^2^ fragments of typical metabolites of 6PPD in positive (B) and negative (C) ESI modes, and MS^2^ fragments of typical metabolites of 6PPD-Q in positive (D) and negative (E) ESI modes.

Biotransformation pathways in *C. elegans* are crucial for understanding contaminant toxicity mechanisms and assessing the nematode’s biodegradation potential, as xenobiotic metabolism can significantly alter a compound’s toxicity. The identified TPs with tentative biological transformation pathways of 6PPD and 6PPD-Q in *C. elegans* are illustrated in Figure 2. Analysis of the pathways of 6PPD implied that it may undergo sequential enzymatic transformations catalyzed by glutathione S-transferases (GSTs), γ-glutamyl transferases (GGTs), UDP-glycosyltransferases (UGTs), and hexokinases (HKs). These enzymatic reactions could convert 6PPD into multiple conjugates, including cysteinyl-6PPD (6DTP388, *m*/*z* 388.2046), 6PPD glucoside (6DTP431, *m*/*z* 431.2540), and 6PPD glucoside-6-phosphate (6DTP511, *m*/*z* 511.2204). Among them, 6DTP431 likely arises from UGT-mediated glycosylation at a secondary amine, while the two 6DTP511 isomers reflect phosphorylation of the glucoside conjugates at distinct amine sites by HKs, consistent with phosphoglucoside formation observed for genistein and albendazole in *C. elegans*.^42,44^ Besides that, we also observed the formation of cysteine-conjugated 6PPD (6DTP388), where 6PPD presumably undergoes glutathione conjugation by GSTs at an electrophilic site, followed by sequential degradation via GGT and cysteinyl-glycine dipeptidase, akin to Cyprocide-B metabolism in *C. elegans*.^45^ Similar pathways were noted for the hydrolysis product of 6PPD, 4-hydroxydiphenylamine (4-HDPA, 6DTP185, *m*/*z* 186.0913), yielding Phase II TPs including 4-HDPA glucoside (6DTP348, *m*/*z* 348.1441) and 4-HDPA glucoside-6-phosphate (6DTP428, *m*/*z* 428.1105). The formation of 4-HDPA may result from either *in vivo* biotransformation in *C. elegans* or in medium abiotic transformation, supported by its substantial presence in the exposure medium (Figure S2). Analysis of medium-only controls confirmed the transformation of 6PPD to 4-HDPA in the culture solutions, which aligns with prior studies reporting significant abiotic conversion of 6PPD to 4-HDPA in aqueous environments^46^ and consistent detection of 4-HDPA in zebrafish embryo exposure media.^47^ These findings suggest that 4-HDPA detected in *C. elegans* likely results from the uptake of abiotically formed 4-HDPA and/or internal metabolism of 6PPD, with downstream TPs indicating its further *in vivo* processing.

**Figure 2:**
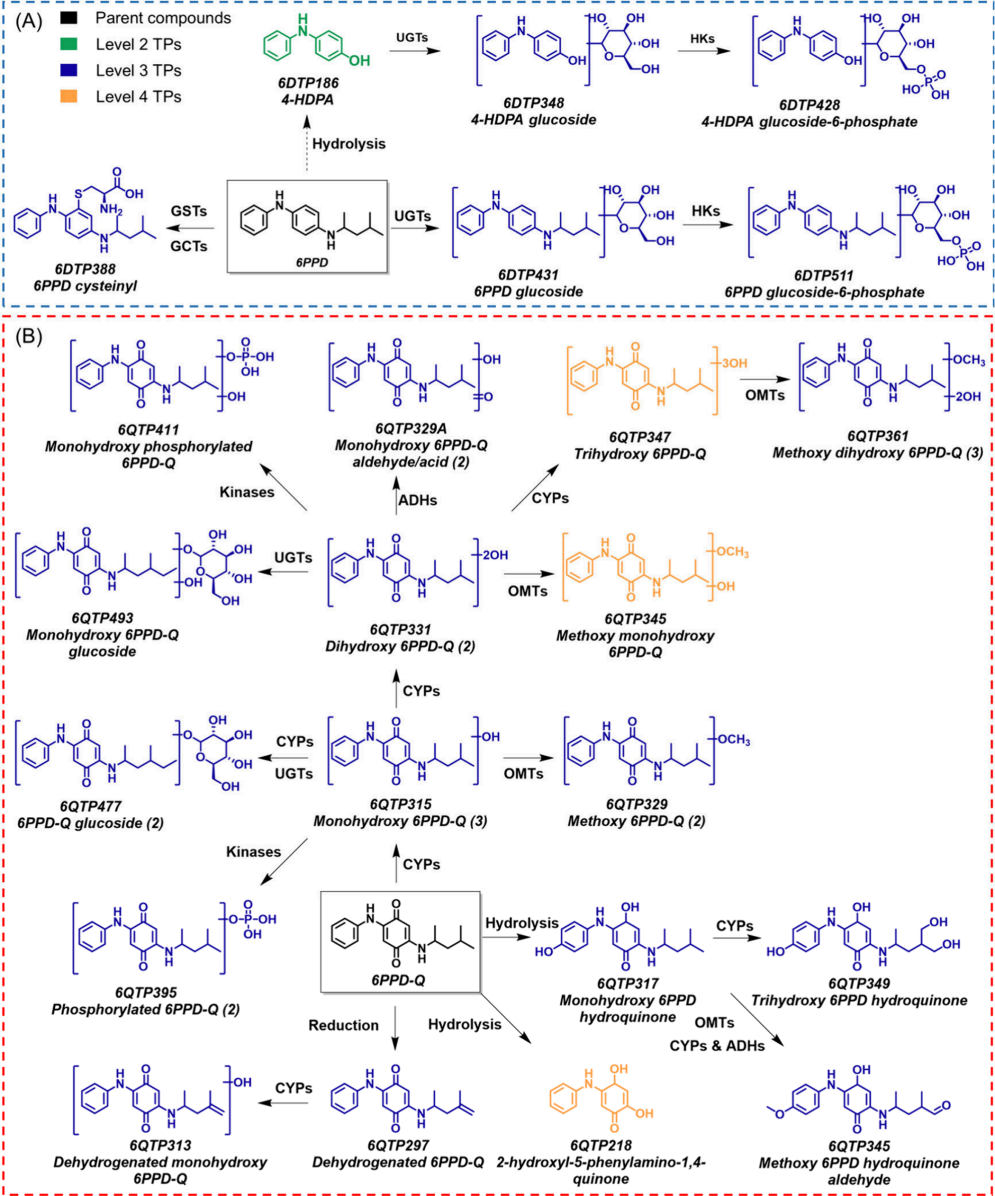
Hypothetical transformation pathways with identified TPs detected in *C. elegans* exposed to 6PPD (A) and 6PPD-Q (B). Reaction pathways are based on the changes in functional groups and putative enzymatic reactions. Molecules with different confidence levels are marked with different colors. GSTs: glutathione S-transferases; GGTs: gamma-glutamyl transferase; UGTs: UDP-glycosyltransferases; HKs: hexokinases; ADHs: alcohol dehydrogenases; CYPs: cytochrome P450; OMTs: O-methyltransferases. The arrows indicate the involved enzymes but not the sequence of the reactions. Dashed arrow represents possible involvement of abiotic transformations in culture medium. The number of isomer quantities is indicated in parentheses, while detailed molecular information on the identified TPs is listed in Table 1.

Regarding 6PPD-Q, 26 TPs were identified in *C. elegans* extracts, including hydroxylated, methoxylated, glycosylated, and phosphorylated metabolites, which could be mediated by alcohol dehydrogenases (ADHs), cytochrome P450 enzymes (CYPs), O-methyltransferases (OMTs), UGTs, and other phosphorylases. Among them, monohydroxy (6QTP315, *m*/*z* 315.1703) and dihydroxy 6PPD-Q (6QTP331, *m*/*z* 331.1652), which were frequently reported in other organisms exposed to 6PPD-Q,^13,16,47–49^ were also detectable in *C. elegans*. The isomeric pattern of the monohydroxy 6PPD-Q (6QTP315), with RTs of 11.57, 13.12, and 14.09 min, reflects diverse hydroxylation sites, consistent with metabolites identified in mice^19^ and in rainbow trout liver cells (RTL-W1).^32^ Moreover, the 6QTP315 isomers could undergo O-methylation to form methoxy 6PPD-Q (6QTP329, *m*/*z* 329.1860), as demonstrated in rice and human *in vitro* models.^50,51^ Other Phase I TPs, including the hydrolysis (6QTP218, *m*/*z* 218.0812) and dehydrogenation product (6QTP297, *m*/*z* 297.1598), which were recently reported in the biotransformation pathways of 6PPD-Q in liver microsome incubations from humans, rats, rainbow trout, and common carp,^49^ were also identified in this study. Mechanistically, dehydrogenation produces 6QTP297 (*m*/*z* 297.1598) which could be formed by removing two hydrogen atoms from 6PPD-Q, while subsequent hydroxylation yields 6QTP313 (*m*/*z* 313.1547). Hydrolysis and reduction of the quinone carbonyl, potentially mediated by quinone reductase, produce 6QTP218 (*m*/*z* 218.0812), followed by hydroxylation on the benzene ring to form 6QTP317 (*m*/*z* 317.1860). This metabolite may then undergo further dihydroxylation to form 6QTP349 (*m*/*z* 349.1758) or methylation and aldehyde formation to generate 6QTP345 (*m*/*z* 345.1809). A variety of Phase II TPs originating from monohydroxy and dihydroxy 6PPD-Q were also observed, including conjugates with pyrophosphate (6QTP395, *m*/*z* 395.1366; 6QTP411, *m*/*z* 411.1316), and glucoside (6QTP477, *m*/*z* 477.2231; 6QTP493, *m*/*z* 493.2181). These biotransformation products were not only recognized in zebrafish embryos^47^ but also recently detected in human urine.^48^ Such observations suggest commonalities in the biotransformation processes of 6PPD-Q in *C. elegans*, humans, other terrestrial mammals, and aquatic organisms. Remarkably, the phosphorylation transformation pathways of both 6PPD-Q (yielding phosphorylated 6PPD-Q of 6QTP395, *m*/*z* 395.1366) and monohydroxy 6PPD-Q (yielding monohydroxy phosphorylated 6PPD-Q of 6QTP411, *m*/*z* 411.1316) were first identified in this study, differing from the sulfation and methylation pathways reported in other organisms.^47–49^ These findings illustrate the novel metabolic pathways of 6PPD-Q in soil organism *C. elegans,* which may inform further efforts to identify contaminant transformations in the soil ecosystem and enhance our understanding of the metabolic mechanism of 6PPD-Q.

The tiny nematode *C. elegans* offers numerous advantages as a model system for discovering and characterizing novel bioactive and biohazard compounds. Compared to xenobiotic metabolism in mammals, *C. elegans* employs distinct enzymatic pathways, such as producing glycosylated derivatives instead of glucuronide conjugates for exogenous toxicants.^42,43,52^ Elucidation of the molecular structures of 6PPD and 6PPD-Q TPs suggests that both UGTs and HKs may play significant roles in the biotransformation of tire-related PPD derivatives. Indeed, previous studies have highlighted the involvement of UGTs and HKs in the metabolic conversion of environmental toxicants, therapeutic drugs, and natural phytoestrogens in *C. elegans*.^44,53^ For example, phase II transformations such as glycosylation are essential for detoxifying benzimidazole anthelmintics such as albendazole in *C. elegans*, where UGT enzymes add a glucose moiety to albendazole to enhance its hydrophilicity.^53^ Soukup et al. also reported that exogenous genistein would undergo stepwise biotransformation by UGTs and HKs to form genistein-7-O-phosphoglucoside in *C. elegans*.^44^ These glycosylated intermediates could potentially undergo further phosphorylation by HKs, which would suggest the importance of UGTs and HKs in the xenobiotic metabolism in *C. elegans*. Given the unique enzymatic transformation pathways and novel TPs of 6PPD and 6PPD-Q identified in *C. elegans*, future research will be essential to evaluate their potential toxicological or detoxification effects on the physiology of *C. elegans*. Notably, while the involvement of specific enzymes in these pathways is reasonably inferred from their known functions, these assignments have not been validated by enzyme activity inhibition experiments. Considering the extensive diversity and redundancy of *C. elegans* enzymes,^54^ each transformation pathway may involve a variable number of enzymatic steps. Further studies are necessary to validate these pathways and clarify the specific roles of the involved enzymes. Such knowledge is critical to explain why specific TPs are formed, to assess the biological consequences of each transformation (e.g., detoxification versus toxification), and to predict how the contaminant’s fate and effects might be influenced by genetic and environmental factors.

### Metabolic Kinetics of TPs in *C. elegans*

Monitoring the reaction kinetics of environmental toxicants in *C. elegans* is crucial for understanding their bioaccumulation, toxicity, and potential impacts on the soil ecosystem. Kinetic analysis revealed that the substrate depletion of 6PPD and 6PPD-Q followed first-order kinetics (Figure 3A). This finding is consistent with 6PPD-Q concentrations observed in zebrafish embryos and the RTL-W1 cell line, where similar elimination patterns were recorded—with most of 6PPD-Q being transformed within 96 h in embryos and within 12 h in the RTL-W1 cell line, respectively.^32,47^ Comparatively, our data suggest that the metabolism of 6PPD and 6PPD-Q in the soil organism *C. elegans* requires a longer duration, exceeding 48 h. A possible interpretation is that the extent of xenobiotic transformation in an organism depends on both compound uptake and the enzymatic capacity.^55^ Kinetic experiments exposing *C. elegans* to either 6PPD or 6PPD-Q showed similar overall temporal trends in the formation of their respective TPs. Excluding TPs present at trace levels below the quantification limit, a total of four 6PPD TPs and 15 6PPD-Q TPs were detected. Based on their time to reach peak concentration (*t*_max_), we classified them into different categories. For 6PPD, TPs showing an accumulative trend over the 24 h monitoring period were classified as 6DTPs-Class I, while the TPs with *t*_max_ < 1 h were classified as 6DTPs-Class II. Similarly, 6PPD-Q TPs exhibiting a continuous accumulation trend were categorized as 6QTPs-Class I, whereas those with *t*_max_ ≤ 3 h and >3 h were classified as 6QTPs-Class II and 6QTPs-Class III, respectively.

**Figure 3:**
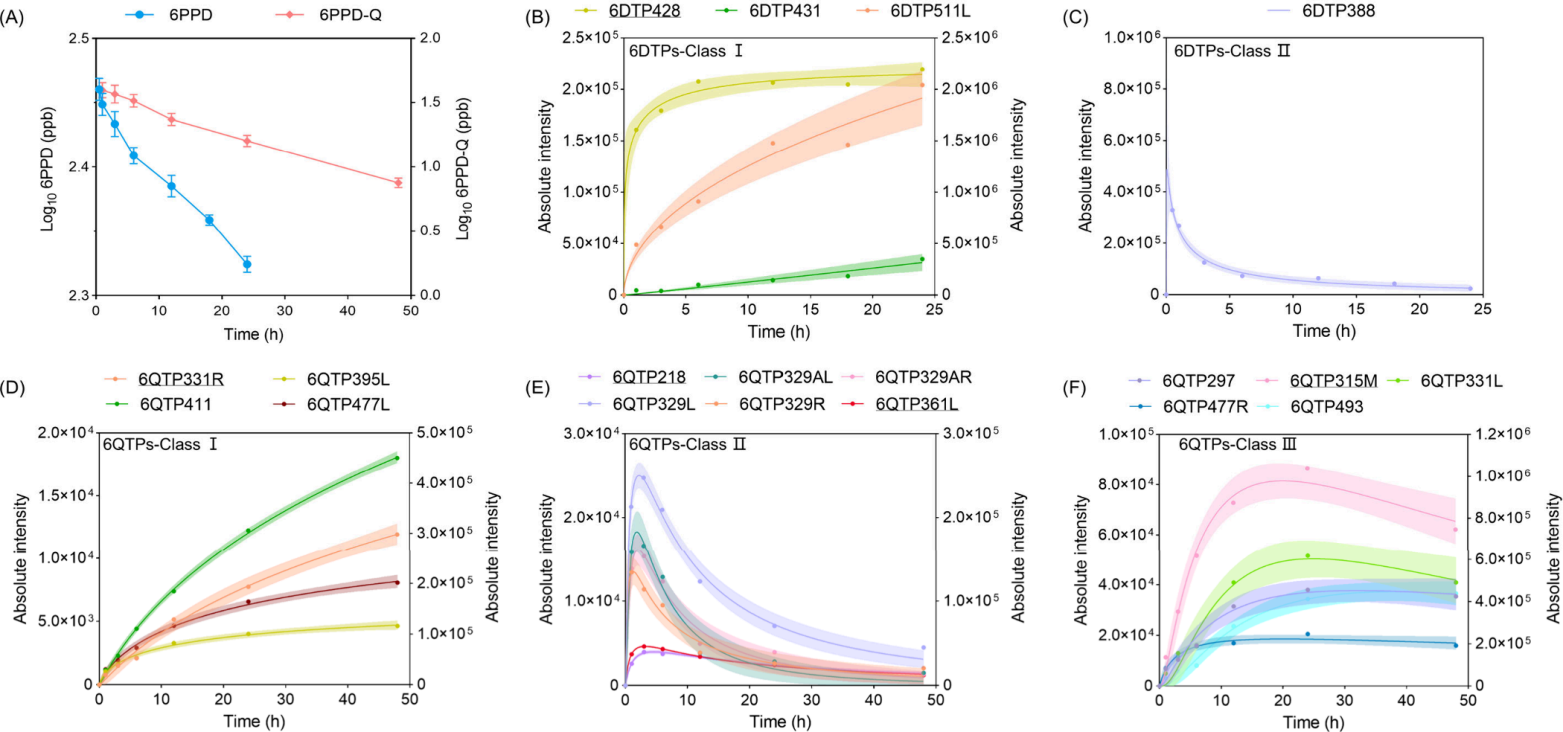
(A) Concentration changes of 6PPD and 6PPD-Q in *C. elegans* extracts over a 48 h period. (B) Abundance changes of 6PPD TPs belonging to Class I (accumulative). (C) Abundance changes of 6PPD TPs belonging to Class II (*t*_max_ < 1 h). (D) Abundance changes of 6PPD-Q TPs belonging to Class I (*t*_max_ > 3 h). (E) Abundance changes of 6PPD TPs belonging to Class II (accumulative). (F) Abundance changes of 6PPD TPs belonging to Class III (*t*_max_ ≤ 3 h). Plotted values are the averages of the measured concentrations with curve fitting using log-normal nonlinear regressions. The colored shadow surrounding the regression plot represents the 95% confidence interval. Three biological replicates were performed at each time interval.

Almost all of the detected 6PPD TPs demonstrated steadily increasing trends during exposure, including 6DTP427, 6DTP431, and 6DTP511 (Figure 3B). A notable exception is cysteinyl 6PPD (6DTP387, Figure 3C), which exhibits a remarkably short *t*_max_ of 0.5 h, followed by a continuous decline over 24 h. Similarly, many 6PPD-Q metabolites, including monohydroxy 6PPD-Q aldehyde (6QTP329A), methoxy 6PPD-Q (6QTP329), methoxy dihydroxy 6PPD-Q (6QTP361R), and 2-hydroxyl-5-phenylamino-1,4-quinone (6QTP218), display a short *t*_max_ (<3 h) as shown in Figure 3E. These TPs, akin to cysteinyl 6PPD that demonstrated a sharp decreasing trend after reaching their *t*_max_, are likely intermediates that undergo further transformation rather than final products. Comparatively, Class III TPs maintain relatively consistent levels after reaching their *t*_max_ (>3 h). Similar transitions, from peak intensities to plateau, were observed for the monohydroxy and dihydroxy 6PPD-Q TPs in RTL-W1 cells.^32^ In contrast, 6QTPs of Class I display continuous accumulation during the exposure period, which includes dihydroxy 6PPD-Q (6QTP331R), phosphorylated 6PPD-Q (6QTP395L), monohydroxy phosphorylated 6PPD-Q (6QTP411), and 6PPD-Q O-glucoside (6QTP477L). Intriguingly, some of these metabolites appear as isomeric forms of Class III 6QTPs, e.g., 6Q331L and 6QTP477R, suggesting that subtle differences in molecular structure can lead to distinct metabolic behaviors. For instance, 6QTP331L and 6QTP331R are identified as dihydroxy 6PPD-Q that differ only in the substitution sites of hydroxyl groups. To explain their divergent transformation kinetics, it was crucial to first confirm their precise structures. In 6QTP331L, one hydroxyl resides at the alkyl-chain terminus and the other on the benzene ring of 6PPD-Q (Figure S8), as evidenced by the characteristic fragments of propanol (C_3_H_7_O^+^, *m*/*z* 59.0491, mass error = 5.1 ppm) and 4-aminophenol (C_6_H_6_NO^+^, *m*/*z* 108.0444, mass error = 5.6 ppm). In contrast, the 1,3-propanediol fragment (C_3_H_7_O_2_^+^, *m*/*z* 75.0447) in 6QTP331R indicates that both hydroxyls are located at the alkyl chain ends. Additional evidence comes from MS^2^ fragment ions of 6QTP331L and 6QTP331R at *m*/*z* 231.0760 (C_12_H_11_N_2_O_3_^+^) and *m*/*z* 241.0968 (C_14_H_13_N_2_O_2_^+^), whose comparative presence confirms a single aromatic-ring hydroxyl in 6QTP331L. Minute differences in substitution sites yield distinct metabolic behaviors: 6QTP331L shows an initial increase followed by stabilization, whereas 6QTP331R exhibits a continuous upward trend. Exposure of 6PPD-Q to RTL-W1 cells revealed similar discrepancies in the dynamic patterns of three dihydroxy 6PPD-Q isomers with different hydroxylation sites: one isomer reached its peak level between 6 and 12 h of exposure, while the others showed continuous accumulative trends throughout the 24 h experiment.^32^ The divergent metabolic behaviors of the isomers indicate that detailed structural characterization is not merely a technical exercise but also a prerequisite for accurately predicting metabolite behavior and stability. Compared to metabolites attributed to Classes II and III that are demonstrated as “intermediate”, those TPs belonging to Class I, from both 6PPD and 6PPD-Q, warrant special attention, as their prolonged stability and accumulation may confer toxicological effects. A recent study reported that 6PPD-Q exhibits a lower bioaccumulation factor (log BAF 1.3–1.9) when compared to 6PPD (log BAF 3.4–4.2).^56^ Given the extensive biotransformation of 6PPD-Q and the accumulative tendencies of its metabolites, further investigation into their occurrence and levels across multiple organisms is justified.

### Toxicity Evaluation of Identified TPs

To evaluate the potential toxicological contributions of the identified TPs, thus explaining the observable toxicity of 6PPD and 6PPD-Q, we further predicted their toxicities using the ECOSAR model developed by USEPA. Multiple organisms were included in the analysis because the *C. elegans* model could serve as a crucial starting point for understanding biotransformation pathways that are likely relevant across different phyla, given the consistent detection of these TPs across various species.^19,32,47–49^ Comparative toxicity profiles of the parent compounds (6PPD/6PPD-Q) and their TPs toward diverse aquatic and terrestrial organisms are presented in Figure 4 and Table S4. Toxicity profiles of TPs diverged significantly from their parent compounds, with distinct patterns observed between the TPs of 6PPD and 6PPD-Q. Except for 6DTP186 (4-HDPA), which exhibited earthworm toxicity comparable to that of 6PPD, all 6PPD metabolites showed a toxicity reduction by at least an order of magnitude, indicating detoxification through biotransformation. In contrast, several biotransformation products of 6PPD-Q illustrate equivalent toxicity scales across all of the evaluated species. These TPs include 6QTP297, 6QTP313, 6QTP315, and 6QTP329A, which involve proposed biological transformations of dehydrogenation, hydroxylation, and carbonylation/carboxylation of 6PPD-Q. Notably, 6QTP315 has been widely detected among multiple species exposed to 6PPD-Q,^19,32,47,57^ while 6QTP313 was also identified to be the metabolite in human liver microsomes and S9 fractions and multiple *in vitro* and *in vivo* models very recently.^48,49^ Given these facts, systematic screening for these biotransformation products in humans and diverse environmental organisms is urgently required. Besides that, the comparable toxicities of particular TPs with 6PPD-Q to earthworms may partially explain the adverse effects of 6PPD-Q to *C. elegans*, necessitating future isolation of these TPs and mechanistic validation through *in vivo* and *in vitro* assays. A recent study examined the *in vitro* CYP-mediated metabolism of 6PPD-Q in human and rat liver microsomes, predicting that multiple Phase I metabolites may exhibit mutagenic and/or immunotoxic effects.^33^ While these predictions establish toxicity benchmarks that can be referenced for novel TPs, rigorous species-specific hazard assessments remain imperative for comprehensive risk characterization.

**Figure 4:**
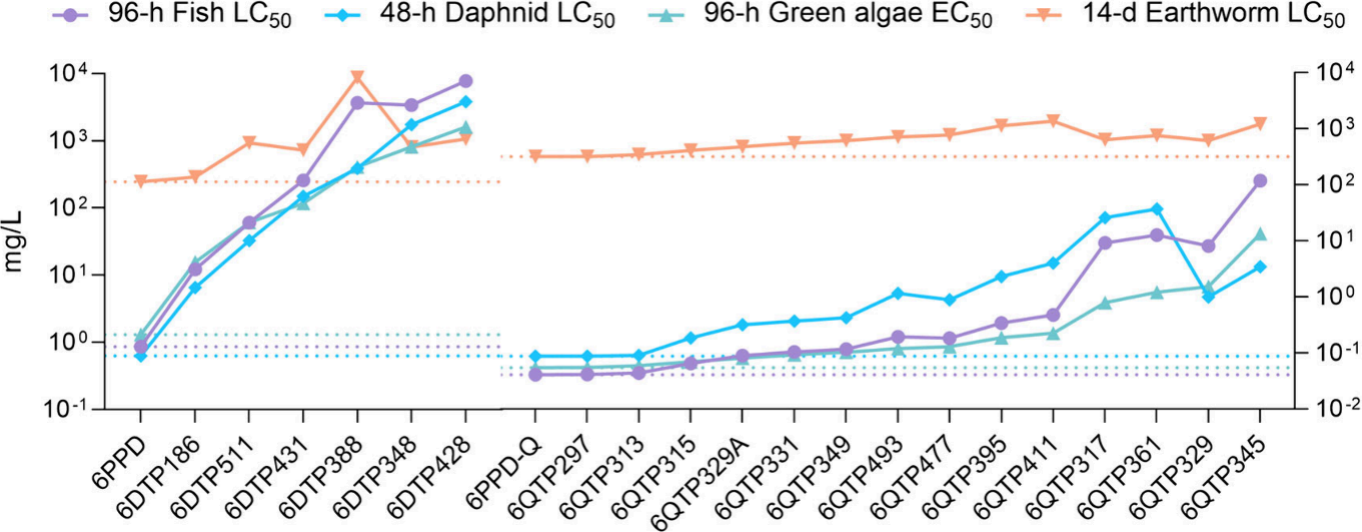
Estimated aquatic toxicity of 6PPD, 6PPD-Q, and their respective biotransformation products. The baseline toxicity of the parent compounds (6PPD and 6PPD-Q) to each species are marked by dotted lines correspondingly.

## Environmental Implications

Biotransformation modulates the toxicity and environmental persistence of commercial product additives, yielding metabolites with altered toxicity. Here, we present comprehensive screening and identify 9 metabolites of 6PPD and 26 TPs of 6PPD-Q in soil model organism *C. elegans*, establishing the first time-resolved metabolic atlas that maps diverse biotransformation pathways and associated metabolites. Notably, novel *in vivo* metabolites—phosphorylated and monohydroxy phosphorylated 6PPD-Q derivatives—were discovered, revealing previously unrecognized detoxification or bioactivation pathways in this terrestrial organism. Semiquantitative kinetic profiling further detailed specific abundance variations of identified TPs over time, highlighting buildup patterns of certain derivatives that warrant prioritized assessment for persistence and environmental/biological occurrence.

Interspecies variability in biotransformation products and kinetics may underlie the pronounced differences in sensitivity and toxicological responses to environmental pollutants, as minor discrepancies in metabolic pathways, TPs, and even minute isomeric structures of the pollutants may lead to significant divergence in their biological properties and even toxicities. Therefore, the establishment of a comprehensive biotransformation data set for tire-derived chemicals, especially 6PPD-Q, which demonstrated species-specific toxicity to particular salmonids, is of great significance. A very recent study revealed that the unique alkyl-OH-6PPD-Q metabolite might play a key role in the toxicity of 6PPD-Q by comparing the different TPs of multiple PPD-Qs in rainbow trout.^31^ Similarly, our study also disclosed the significantly distinct kinetic profiles of isomeric TPs with minor differences in the substitution sites.

Collectively, this study delineates 6PPD and 6PPD-Q biotransformation pathways, identifies novel metabolites, and provides insights into their kinetics and potential toxicity in a soil organism. However, as synthesized standards for the novel TPs identified via HRMS are currently unavailable, further confirmation of their specific structures is necessary, as constitutional and stereoisomerism can significantly affect bioactivity.^15^ While this study provides a hypothesis-generating data set mapping TPs and kinetics, future research employing synthesized standards for *in vivo* and *in vitro* toxicological validation, alongside genetic tools (e.g., RNAi, mutant strains) to confirm enzyme roles, is essential. Given the increasing environmental presence of structurally analogous p-phenylenediamines (PPDs) and their quinones (PPD-Qs) in soils,^6^ large-scale screening of their TPs represents a promising avenue for discovering novel biomarkers to monitor contamination in environmental matrices and ecological communities.

## Supplementary Material



## Notes

Additional description about the exposure concentration estimation and selection of single-dose exposure (Text S1); suspect and nontargeted screening analysis (Text S2); calculation of matrix effects (Text S3); instrument parameters used in the quantification and qualification of the analytes (Table S1); optimized MRM parameters (Table S2); in-house screening list of biotransformation products of 6PPD and 6PPD-Q (Table S3); predicted ecotoxicities of 6PPD, 6PPD-Q, and their biological TPs (Table S4); statistics of lethality of *C. elegans* during exposure to 6PPD and 6PPD-Q (Figure S1); external concentration of 6PPD, 6PPD-Q, and 4-HDPA in the culture (Figure S2); Calibration curves used for the determination of 6PPD and 6PPD-Q (Figure S3); MS/MS fragments of the identified biological transformation products and their structure elucidations (Figures S4–S11) (PDF)
