# Supplementary material for: Biotransformation of Tire-Derived 6PPD and 6PPD‑Q in Soil Nematode Caenorhabditis elegans: Unraveling Novel Phosphorylation Products and Distinct Kinetic Profiles
Source: Environ Sci Technol. 2025 Jul 8;59(28):14625–36. doi: 10.1021/acs.est.5c02072 (PMC12288073; doi:10.1021/acs.est.5c02072)
Supplement: Supplementary file 1 [file es5c02072_si_001.pdf]

Supporting Information

Biotransformation of Tire-Derived 6PPD and 6PPD-Q in Soil Nematode *Caenorhabditis elegans*: Unraveling Novel Phosphorylation Products and Distinct Kinetic Profiles

Wei Wang[a,1], Gefei Huang[a,1], Fangfang Miao[a], Zhongying Zhao[b], and Zongwei Cai[a, c, *]

[a] *State Key Laboratory of Environmental and Biological Analysis, Department of Chemistry, Hong Kong Baptist University, Hong Kong SAR 999077, China*

[b] *Department of Biology, Hong Kong Baptist University, Hong Kong SAR 999077, China*

[c] *Eastern Institute of Technology, Ningbo 315200, China*

Number of pages: 34

Number of figures: 11

Number of tables: 4

[*] Corresponding author: Zongwei Cai, email: zwcai@hkbu.edu.hk

[1] Both authors contributed equally to this work.

Contents

Text S1. Exposure concentration estimation and selection of single-dose exposure.........................3

Text S2. Additional description about the suspect and nontargeted screening analysis....................5

Text S3. Calculation of matrix effects of the culture medium..7

Table S1. Instrument parameters used in the qualification and quantification of the analytes..........8

Table S2. Optimized MRM parameters for the semi-quantification of the biological TPs...............9

Table S3. In-house screening list of biotransformation products of 6PPD and 6PPD-Q 20

Table S4. Predicted ecotoxicities of 6PPD, 6PPD-Q and their biological TPs 21

Figure S1. Statistics of lethality of *C. elegans* during exposure to 6PPD and 6PPD-Q 22

Figure S2. External concentration of 6PPD, 6PPD-Q, and 4-HDPA in the culture 23

Figure S3. Calibration curves used for the determination of 6PPD and 6PPD-Q 26

Figure S4. MS/MS fragments of 6DTP388, 6DTP431, 6DTP511R, and 6DTP186. 25

Figure S5. MS/MS fragments of 6DTP348L, 6DTP348R, 6DTP428L (+ and -ESI)......................26

Figure S6. MS/MS fragments of 6DTP428R...27

Figure S7. MS/MS fragments of 6QTP297, 6QTP313, 6QTP315L and 6QTP315M.....................28

Figure S8. MS/MS fragments of 6QTP329AL, 6QTP329AL, 6QTP331L, and 6QTP331R..........29

Figure S9. MS/MS fragments of 6QTP349, 6QTP477L, 6QTP477R, and 6QTP493....................30

Figure S10. MS/MS fragments of 6QTP395L, 6QTP317, 6QTP361R, and 6QTP329L 31

Figure S11. MS/MS fragments of 6QTP345R...32

References...33

Text S1. Exposure concentration estimation and selection of single-dose exposure.

The exposure designs are based on the rapid lifecycle and small size of *C. elegans*, which necessitate shorter exposures and higher exposure concentrations compared to other model organisms. The lifecycle of *C. elegans* (~3 days at 20°C) is significantly faster,[1] enabling acute exposures of 24–48 hours to capture peak biotransformation during the L4 stage. Its small size and biomass (~1 mm, ~10 mg/10,000 worms per 10 mL replicate) limit chemical uptake, requiring a high concentration (100 μg/L) to detect TPs via UPLC-HRMS.

Exposure concentration estimation. Higher concentrations affect the survival and biological functions, reducing *C. elegans'* capacity to metabolize 6PPD and 6PPD-Q, while lower concentrations enhanced survival but yielded insufficient substrate for detectable TP formation. We thus tested concentrations from 10 $\mu g \cdot L^{-1}$ to 200 $\mu g \cdot L^{-1}$ and monitored lethality over the exposure period. Although 10 $\mu g \cdot L^{-1}$ was non-lethal to L4 worms for both 6PPD and 6PPD-Q, the biotransformation products, particularly those of lower abundance, were not effectively detected by our UPLC-MS platform. At 50 $\mu g \cdot L^{-1}$ and 100 $\mu g \cdot L^{-1}$, slight lethality emerged after 24 hours of exposure, yet no significant difference in lethality was observed between these concentrations. Worms exposed to 200 $\mu g \cdot L^{-1}$ of 6PPD and 6PPD-Q show significant lethality after 24 hours of exposure. Consequently, we adopted 100 $\mu g \cdot L^{-1}$ as the exposure concentration for both testing compounds, as a trade-off between biotransformation degrees and the physiological activity of worms. Higher concentrations were avoided due to elevated lethality and concerns about exceeding the aqueous solubility limits of these compounds, particularly 6PPD-Q.

Selection of single dose exposure. The single dose exposure is a widely adopted method for toxicant exposures in *C. elegans* due to its practical advantages and alignment with the organism's biological traits. Many previous studies adopted such methods to investigate the toxicological and metabolic effects of contaminants in *C. elegans,*[2-4] and the biotransformation of 6PPD-Q in other organisms.[5-7] In addition to the normal manipulation of single-dose exposure, tire-derived chemicals, including 6PPD and 6PPD-Q follow the exposure of episodic nature. Previous studies indicated that during the Pacific Northwest wet season, regular intervals of rainfall wash 6PPD-Q off roads to both streams and soils.[8] In contrast, during the summer dry season, 6PPD-Q deposition builds up on roads. This wet/dry cycle creates discrete exposures of 6PPD-Q delivery to streams, not a continuous input. The same pulse increases of 6PPD have also been proposed by Hiki et. al, who found that 6PPD

leaches rapidly from dust into water (declining within 4 hours), which suggests a short, intense exposure during rainfall events rather than sustained release.[9] Therefore, we did not select a constant level of 6PPD and 6PPD-Q exposure in our current study.

Text S2. Additional description about the suspect and nontargeted screening analysis.

Suspect screening. Suspect screening enables highly efficient identification of biotransformation products through systematic integration of published transformation mechanisms and functional group alteration patterns documented in established databases. In this study, we integrated dual approaches to construct a comprehensive in-house screening database. By compiling previously reported metabolites of 6PPD and 6PPD-Q from model organisms (zebrafish,[10] mice,[11] and lettuce [12]) and leveraging computational tools (EAWAG-BBD Pathway Prediction System [13] and Biotransformer 3.05 [14]), we constructed a customized metabolite candidate database comprising 226 potential transformation products (Table S5). This integrated screening strategy not only enhanced the detection efficiency of biotransformation products but also revealed mechanistic patterns underlying their metabolic pathways.

The EAWAG-BBD system encompasses 249 biotransformation rules with 332 annotated reaction descriptions. All system-inherent biotransformation rules were applied, generating products with complete carbon skeleton retention unless explicitly modified by reaction logic. Biotransformer 3.0 employs a Metabolism Prediction Tool (BMPT) architecture with six specialized modules: EC-based transformations, CYP450-mediated Phase I reactions, Phase II conjugation, human gut microbial metabolism, environmental microbial degradation, and abiotic transformation processes. All modules were utilized to simulate potential transformation pathways of 6PPD and 6PPD-Q, ensuring comprehensive coverage of biological and environmental metabolic scenarios. Key parameters were optimized as follows: reaction iteration depth = 3, CYP450 reaction mode = 'combined' (incorporating both experimental and predicted enzymatic activities).

Nontargeted screening. To identify potential unknown biotransformation products, especially unique metabolites of 6PPD and 6PPD-Q in *C elegance*, we also adopted nontargeted screening analysis methods. Generally, the experimental design comprised three distinct groups: *C. elegans*-only (Group A), contaminant-only (6PPD/6PPD-Q, Group B), and co-exposure (*C. elegans* + contaminants, Group C). Novel biotransformation products were identified by comparative analysis of Group C against Groups A and B. Specifically, chromatographic peaks unique to Group C or with abundance ten times higher were isolated by subtracting the full-scan chromatograms of Groups A and B. High-resolution mass spectrometry provided accurate molecular masses (<5 ppm error) and isotopic patterns to determine elemental compositions of newly detected molecules. Their

molecular formulas were compared with their parent compounds (6PPD or 6PPD-Q). Functional group alterations (e.g., hydroxylation, phosphorylation) were deduced from mass differences ($\Delta m/z$) and used to hypothesize plausible transformation pathways and structures. To validate structural assignments, scheduled parallel reaction monitoring (PRM) was performed across normalized collision energies (NCEs) ranging from 10% to 40%, generating diagnostic MS/MS spectra. Fragmentation patterns were analyzed to infer substructures, focusing on characteristic fragment ions (e.g., neutral losses of H_3PO_4 for phosphorylated derivatives) and cleavage patterns including β-cleavage, Retro-Diels-Alder cleavage, McLafferty rearrangement and aromatic rearrangement (Figure S4-S11).

Text S3. Calculation of matrix effects of the culture medium.

Matrix effects (MEs) were evaluated by spiking 6PPD and 6PPD-Q into the extracts and cultures, respectively. We evaluate the matrix effect by comparing the abundance of compounds in the matrix compared to the abundance in pure solvents. To achieve this, we spiked an identical volume of standards of 6PPD and 6PPD-Q into the extracts and medium, respectively, and compared their responses to the pure standards. The calculation equations are as follows:

$$ME\ (\%) = \frac{Abu_{MS} - Abu_{MU}}{Abu_{SPS}} \times 100$$

Among them, ME is the calculated matrix effects (%); Abu_{MS} is the abundance of the analytes in the culture medium with specific spiking volumes of the analytes; Abu_{MU} is the abundance of the analytes in the culture medium without spiked; Abu_{SPS} is the abundance of the analytes in the pure solvent. Three concentration levels (10, 50, and 100 $\mu g \cdot L^{-1}$) were tested in triplicate. The resulting ME for 6PPD was 101 ± 9% in cultures and 106 ± 13% in extracts, while for 6PPD-Q it was 102 ± 4% in cultures and 90 ± 5% in extracts.

Table S1. Instrument parameters used in the quantification and qualification of the analytes.

Instrument	Parameter	Value
UPLC	Flow rate	300 µL·min^{-1}
	Injection volume	2 µL
	Column	Waters Acquity HSS T3 column (1.8 µm, 2.1×100 mm)
	Column temperature	35 ℃
	Mobile phase	(A) deionized water with 0.1% formic acid in (B) acetonitrile with 0.1% formic acid
	Solvent gradient	2% B for 1 min, increased linearly to 100% B in 19 min and hold for 3 min, decreased to 2% B in 0.1 min and hold for 4.9 min.
ESI-Q Orbitrap MS	Ionization mode	Positive ESI
	Vaporizer temperature	300 ℃
	Spray voltage	3500 V
	Scan range	80-500
	Sheath gas flow	45 arb
	Auxiliary gas flow	10 arb
	Cone gas	Nitrogen
	Collision gas	Argon
	Full MS resolution	35,000 FWHM
	ddMS^2 resolution	17,500 FWHM
	PRM NCE	10 20 30 40%
ESI-TQ MS	Ionization mode	Positive ESI
	Vaporizer temperature	350 ℃
	Cone gas	Nitrogen
	Collision gas	Argon
	Collision energy	Specified for each analyte (Table S3)

Table S2. Optimized MRM parameters for the semi-quantification of the biological transformation products.

Compound	Mode	Precursor ion	Quantifier product ion	Collision energy (eV)	Qualifier product ion 1	Qualifier product ion 2
6PPD	+MRM	269.2	93.1	32	184.1	212.1
6DTP428	-MRM	426.1	134.0	20	179.0	358.1
6DTP431	+MRM	431.3	120.0	12	86.1	166.1
6DTP511	-MRM	509.2	222.0	13	294.1	473.3
6DTP388	+MRM	388.2	166.1	14	86.1	268.2
6PPD-Q	+MRM	299.2	241.1	26	215.1	187.1
6QTP331R	+MRM	331.2	215.1	15	257.1	295.1
6QTP395L	+MRM	395.1	297.2	16	241.1	215.1
6QTP411	+MRM	411.1	241.9	15	295.1	393.1
6QTP477L	+MRM	477.2	315.2	15	215.1	99.1
6QTP218	-MRM	216.1	100.0	18	170.1	198.1
6QTP329AL	+MRM	329.2	311.1	11	241.1	200.1
6QTP329AR	+MRM	329.2	200.1	15	238.1	311.1
6QTP329L	+MRM	329.2	257.1	15	202.1	62.1
6QTP329R	+MRM	329.2	152.1	13	245.1	293.1
6QTP361R	+MRM	361.2	102.1	21	232.1	260.1
6QTP297	+MRM	297.2	241.1	15	215.1	83.1
6QTP315M	+MRM	315.2	231.1	11	273.1	85.1
6QTP331L	+MRM	331.2	257.1	15	273.1	203.1
6QTP477R	+MRM	477.2	231.1	25	210.2	315.2
6QTP493	+MRM	493.2	257.1	25	231.1	419.1

Table S3. In-house screening list of biotransformation products of 6PPD and 6PPD-Q for suspect screenings.

Category	Name/Reaction pathway	*m/z* (+)	*m/z* (-)	RT	Source
6PPD TPs	4-HDPA+Cys+glucuronide	481.1276	479.1125	5.91	Nico Grasse et al.[10]
6PPD TPs	4-HDPA quinonimine + O +SO3		278.0131	4.66	
6PPD TPs	4-HDPA + 2O	218.0817	216.0654	4.56	
6PPD TPs	6-PPD+O+glucuronide	461.2275	459.213	5.74	
6PPD TPs	4-HDPA+2O+CH3	232.0965		6.07	
6PPD TPs	6-PPD+O+glucuronide +glutathione	764.2814	762.2663	6.26	
6PPD TPs	4-HDPA+NAcCys+glutathione	650.1590	648.1440	6.63	
6PPD TPs	6-PPD+O+Cys	404.1988	402.1844	6.68	
6PPD TPs	4-HDPA +glucuronide +CH2O (Acyl)	392.1343	390.1188	6.77	
6PPD TPs	4-HDPA+O+acyl-glucuronide+NAcCys	583.1590	581.1439	6.89	
6PPD TPs	4-HDPA+GSH+NAcCys	650.1590	648.1440	7.07	
6PPD TPs	4-HDPA+SO3	266.0480	264.034	7.08	
6PPD TPs	6-PPD+O+NAcCys	446.2105	444.1955	7.71	
6PPD TPs	4-HDPA	186.0920		7.94	
6PPD TPs	4-HDPA+NAcCys	345.0920	343.0755	7.98	
6PPD TPs	6-PPD+Cys	388.2046	386.1896	8.06	
6PPD TPs	6-PPD+glutathione	572.2549	570.2386	8.13	
6PPD TPs	4-HDPA+Cys-Gly+Acety	407.1260	405.1123	8.32	
6PPD TPs	6-PPD+NAcCys	430.2148		8.83	
6PPD-Q TPs	6-PPDQ+O+glucuronide	491.2030	489.1884	10.98	

6PPD-Q TPs	6-PPDQ+O+SO3	395.1263	393.118	8.96	
6PPD-Q TPs	6-PPDQ+2 O+glucuronide	507.1978	505.1826	9.47	
6PPD-Q TPs	6-PPDQ+2O+SO3	411.1220	409.1063	6.83	
6PPD-Q TPs	6-PPDQ+O+GSH	620.2386	618.222	7.23	
6PPD-Q TPs	6-PPDHQ+acylglucuronide+NAcCys	682.2278	680.2127	8.62	
6PPD-Q TPs	6-PPDQ+2O+CH3+Cys	464.1835	162.1694	8.87	
6PPD-Q TPs	6-PPDQ+O+NAcCys	476.1846		9.03	
6PPD-Q TPs	6-PPDQ+O	315.1702		9.68	
6PPD TPs	6-PPD+Glucose Conjugation	431.2516		5.3	
6PPD-Q TPs	6-PPDQ_TP262	262.1429		9.5	
6PPD-Q TPs	6-PPDQ_TP174	174.1277		9.4	
6PPD-Q TPs	6-PPDQ_TP214	214.2165		9.6	
6PPD-Q TPs	C12H11N2O2	215.0815			
6PPD-Q TPs	C6H11O	99.0804			Stephanie Castan et al.[12]
6PPD-Q TPs	C18H25N2O2	301.1911			
6PPD-Q TPs	C12H10NO3	216.0655			
6PPD-Q TPs	C18H23N2O3	315.1703			
6PPD-Q TPs	C6H7O2	111.0441			
6PPD-Q TPs	C12H19N2O2	223.1441			
6PPD-Q TPs	C18H25N2O2	301.1911			
6PPD-Q TPs	C18H21N2O2	297.1598			
6PPD-Q TPs	GSH-conjugation of alpha, beta-unsaturated carbonyl	604.2436			

6PPD-Q TPs	GSH-conjugation of quinone	606.2592	
6PPD-Q TPs	N-Dehydrogenation of aliphatic azaheterocyclene	313.1547	
6PPD-Q TPs	4-Dephosphorylation of phosphatidylinositol-4,5-bisphosphate	317.186	
6PPD-Q TPs	7-OH-Sulfation of sterol	295.1441	
6PPD-Q TPs	4-Dephosphorylation of phosphatidylinositol-4,5-bisphosphate	217.0972	
6PPD-Q TPs	Hydroxylation of non-terminal aliphatic carbon adjacent to aromatic ring	231.0764	
6PPD-Q TPs	7-OH-Sulfation of sterol	329.1496	
6PPD-Q TPs	N-Glucuronidation of hydroxylamine	347.1601	
6PPD-Q TPs	7-OH-Sulfation of sterol	311.139	
6PPD-Q TPs	4-Dephosphorylation of phosphatidylinositol-4,5-bisphosphate	333.1809	
6PPD TPs	Deacetylation of arylacetamide	285.1961	EAWA
6PPD TPs	EAWAG_RULE_BT0350_PATTERN2	267.1856	G-
6PPD TPs	N-Dealkylation of secondary arylalkylamine AndFromCyProduct	99.0804	BBD
6PPD TPs	EAWAG_RULE_BT0023_PATTERN1	185.1073	Pathwa
6PPD TPs	EAWAG_RULE_BT0416_PATTERN1	283.1805	y
6PPD TPs	p-Hydroxylation of phenol	301.1911	Predict
6PPD TPs	N-Dealkylation of secondary arylalkylamine AndFromCyProduct	115.0754	ion
6PPD TPs	Ozonation secondary amines_M2	265.1699	System
6PPD TPs	N-Dealkylation of secondary arylalkylamine AndFromCyProduct	201.1022	and
6PPD TPs	Reduction of quinone	299.1759	Biotran
6PPD TPs	Hydroxylation of non-terminal aliphatic carbon adjacent to aromatic ring	281.1648	sforme
6PPD TPs	p-Hydroxylation of phenol	317.1860	r 3

6PPD TPs	Ozonation secondary amines_M3	131.0703	system
6PPD TPs	N-Dealkylation of secondary arylalkylamine AndFromCyProduct	217.0972	[13, 14]
6PPD TPs	N-Acetylation of arylamine	215.0815	
6PPD TPs	Aromatic OH-glucuronidation	61.02842	
6PPD TPs	EAWAG_RULE_BT0437_PATTERN2	94.06515	
6PPD TPs	Sulfation of primary alcohol	99.08046	
6PPD TPs	2-Hydroxylation of 1,4-disubstituted benzene	101.0961	
6PPD TPs	Ozonation secondary amines_M2	110.06	
6PPD TPs	EAWAG_RULE_BT0350_PATTERN2	110.06006	
6PPD TPs	SNP-Oxidation from CyProduct	115.0753	
6PPD TPs	p-Hydroxylation of phenol	116.107	
6PPD TPs	Ozonation secondary amines_M2	117.091	
6PPD TPs	Catechol O-methylation	124.03933	
6PPD TPs	N-Acetylation of arylamine	126.0549	
6PPD TPs	Chlorination of aliphatic amines_Pattern1	126.05498	
6PPD TPs	N-Hydroxylation of secondary arylamide	132.1019	
6PPD TPs	Terminal desaturation	133.0859	
6PPD TPs	7-OH-Sulfation of sterol	136.0757	
6PPD TPs	Glucuronidation of primary aromatic amine	152.0706	
6PPD TPs	SNP-Oxidation from CyProduct	185.1073	
6PPD TPs	Oxidation of primary alcohol to aldehyde	197.0478	
6PPD TPs	N-Glucuronidation of hydroxylamine	201.1022	

6PPD TPs	Aromatic OH-glucuronidation	207.1492
6PPD TPs	Alkyl-OH-glucuronidation	211.1567
6PPD TPs	SNP-Oxidation from CyProduct	215.08153
6PPD TPs	SNP-Oxidation from CyProduct	217.0971
6PPD TPs	Diphenylamine Photocyclization to Carbazole	223.1441
6PPD TPs	Hydroxylation of terminal methyl	225.1723
6PPD TPs	Hydroxylation from CyProduct	227.1179
6PPD TPs	Chlorination of aliphatic amines_Pattern1	241.1102
6PPD TPs	EAWAG_RULE_BT0337_PATTERN6	243.1128
6PPD TPs	Catechol O-methylation	265.1699
6PPD TPs	2-Hydroxylation of 1,4-disubstituted benzene	267.1855
6PPD TPs	Chlorination of aliphatic amines_Pattern1	270.0972
6PPD TPs	Chlorination of aliphatic amines_Pattern1	281.16486
6PPD TPs	Chlorination of aliphatic amines_Pattern1	283.1805
6PPD TPs	Glucuronidation of primary aromatic amine	285.1961
6PPD TPs	Sulfation of primary alcohol	286.0921
6PPD TPs	Sulfation of primary alcohol	293.1231
6PPD TPs	Aromatic OH-glucuronidation	299.17543
6PPD TPs	Sulfation of primary alcohol	300.18325
6PPD TPs	Alkyl-OH-glucuronidation	301.1466
6PPD TPs	Aromatic OH-glucuronidation	301.191
6PPD TPs	Alkyl-OH-glucuronidation	301.19108

6PPD TPs	GSH-conjugation of epoxide	303.1622
6PPD-Q TPs	N-Dealkylation of secondary arylalkylamine	94.06515
6PPD-Q TPs	EAWAG_RULE_BT0437_PATTERN1	99.08046
6PPD-Q TPs	N-Dealkylation of acyclic secondary amine AndFromCyProduct	100.11209
6PPD-Q TPs	EAWAG_RULE_BT0437_PATTERN1	101.0961
6PPD-Q TPs	N-Dealkylation of acyclic secondary amine AndFromCyProduct	102.1277
6PPD-Q TPs	N-Dealkylation of secondary arylalkylamine	110.06
6PPD-Q TPs	p-Hydroxylation of phenol	110.06006
6PPD-Q TPs	EAWAG_RULE_BT0065_PATTERN1	111.044
6PPD-Q TPs	EAWAG_RULE_BT0437_PATTERN1	115.0753
6PPD-Q TPs	Ozonation secondary amines_M2	116.107
6PPD-Q TPs	EAWAG_RULE_BT0437_PATTERN1	117.091
6PPD-Q TPs	N-Dealkylation of acyclic secondary amine AndFromCyProduct	118.1226
6PPD-Q TPs	Hydroxylation from CyProduct	118.12266
6PPD-Q TPs	Ozonation secondary amines_M3	132.1019
6PPD-Q TPs	Degradation of nitrile to carboxamide	133.0859
6PPD-Q TPs	Aromatic OH-glucuronidation	136.0757
6PPD-Q TPs	N-Dealkylation of secondary arylalkylamine	140.0342
6PPD-Q TPs	Sulfation of primary alcohol	197.0478
6PPD-Q TPs	EAWAG_RULE_BT0437_PATTERN1	215.0815
6PPD-Q TPs	N-Dealkylation of acyclic secondary amine AndFromCyProduct	216.0655
6PPD-Q TPs	4-Dephosphorylation of phosphatidylinositol-4,5-bisphosphate	217.0971

6PPD-Q TPs	N-Dealkylation of acyclic secondary amine AndFromCyProduct	218.0811
6PPD-Q TPs	4-Dephosphorylation of phosphatidylinositol-4,5-bisphosphate	219.1128
6PPD-Q TPs	N-Dealkylation of secondary arylalkylamine	222.1124
6PPD-Q TPs	EAWAG_RULE_BT0065_PATTERN1	223.1441
6PPD-Q TPs	N-Dealkylation of secondary arylalkylamine	224.1281
6PPD-Q TPs	N-Dealkylation of secondary arylalkylamine	226.1437
6PPD-Q TPs	Hydroxylation of non-terminal aliphatic carbon adjacent to aromatic ring	231.07644
6PPD-Q TPs	N-Dealkylation of acyclic secondary amine AndFromCyProduct	232.0604
6PPD-Q TPs	Hydroxylation from CyProduct	232.06046
6PPD-Q TPs	4-Dephosphorylation of phosphatidylinositol-4,5-bisphosphate	233.092
6PPD-Q TPs	p-Hydroxylation of phenol	233.09209
6PPD-Q TPs	Ozonation secondary amines_M2	237.1233
6PPD-Q TPs	Ozonation secondary amines_M2	239.139
6PPD-Q TPs	N-Dealkylation of secondary arylalkylamine	240.123
6PPD-Q TPs	Hydroxylation from CyProduct	240.12306
6PPD-Q TPs	1-Acyl hydrolysis of phosphatidylcholine	247.0713
6PPD-Q TPs	Hydroxylation of non-terminal aliphatic carbon adjacent to aromatic ring	247.07136
6PPD-Q TPs	Ozonation Phenolate or aniline pathway_3_O	248.0553
6PPD-Q TPs	Ozonation Phenolate or aniline pathway_3_O	250.071
6PPD-Q TPs	Ozonation secondary amines_M2	253.1183
6PPD-Q TPs	Ozonation Phenolate or aniline pathway_3_O	258.1336
6PPD-Q TPs	Ozonation Phenolate or aniline pathway_3_O	266.0659

6PPD-Q TPs	Glucuronidation of primary aromatic amine	270.0972
6PPD-Q TPs	Chlorination of aliphatic amines_Pattern1	271.0844
6PPD-Q TPs	Ozonation Phenolate or aniline pathway_3_O	274.1285
6PPD-Q TPs	Ozonation Phenolate or aniline pathway_3_O	284.032
6PPD-Q TPs	Chlorination of aliphatic amines_Pattern1	292.0946
6PPD-Q TPs	Alkyl-OH-glucuronidation	293.1231
6PPD-Q TPs	7-OH-Sulfation of sterol	295.1441
6PPD-Q TPs	Sulfation of secondary alcohol	297.0539
6PPD-Q TPs	7-OH-Sulfation of sterol	297.1597
6PPD-Q TPs	4-Dephosphorylation of phosphatidylinositol-4,5-bisphosphate	299.1754
6PPD-Q TPs	N-Demethylation of aryldimethylamine	299.17543
6PPD-Q TPs	4-Dephosphorylation of phosphatidylinositol-4,5-bisphosphate	301.191
6PPD-Q TPs	4-Dephosphorylation of phosphatidylinositol-4,5-bisphosphate	303.2067
6PPD-Q TPs	Reduction of carbonyl	305.2223
6PPD-Q TPs	EAWAG_RULE_BT0107	307.238
6PPD-Q TPs	7-OH-Sulfation of sterol	311.139
6PPD-Q TPs	Hydroxylation of non-terminal aliphatic carbon adjacent to aromatic ring	311.13904
6PPD-Q TPs	N-Dehydrogenation of aliphatic azaheterocyclene	313.1546
6PPD-Q TPs	Hydroxylation of non-terminal aliphatic carbon adjacent to aromatic ring	313.15469
6PPD-Q TPs	EAWAG_RULE_BT0429	315.1703
6PPD-Q TPs	Hydroxylation of non-terminal aliphatic carbon adjacent to aromatic ring	315.17034
6PPD-Q TPs	4-Dephosphorylation of phosphatidylinositol-4,5-bisphosphate	317.1859

6PPD-Q TPs	Hydroxylation of non-terminal aliphatic carbon adjacent to aromatic ring	317.18599
6PPD-Q TPs	4-Dephosphorylation of phosphatidylinositol-4,5-bisphosphate	319.2016
6PPD-Q TPs	p-Hydroxylation of phenol	319.20164
6PPD-Q TPs	Aldehyde oxidation	329.1496
6PPD-Q TPs	Hydroxylation of non-terminal aliphatic carbon adjacent to aromatic ring	329.14961
6PPD-Q TPs	EAWAG_RULE_BT0429	331.1652
6PPD-Q TPs	Hydroxylation of non-terminal aliphatic carbon adjacent to aromatic ring	331.16526
6PPD-Q TPs	Chlorination of aliphatic amines_Pattern1	333.1364
6PPD-Q TPs	4-Dephosphorylation of phosphatidylinositol-4,5-bisphosphate	333.1809
6PPD-Q TPs	Hydroxylation of non-terminal aliphatic carbon adjacent to aromatic ring	333.18091
6PPD-Q TPs	Chlorination of aliphatic amines_Pattern1	335.1521
6PPD-Q TPs	Catechol O-methylation	345.1809
6PPD-Q TPs	N-Glucuronidation of hydroxylamine	347.1601
6PPD-Q TPs	Hydroxylation of non-terminal aliphatic carbon adjacent to aromatic ring	347.16017
6PPD-Q TPs	Chlorination of aliphatic amines_Pattern1	349.1313
6PPD-Q TPs	Ozonation Phenolate or aniline pathway_3_O	349.1758
6PPD-Q TPs	Chlorination of aliphatic amines_Pattern1	351.147
6PPD-Q TPs	Chlorination of aliphatic amines_Pattern1	367.0974
6PPD-Q TPs	Chlorination of aliphatic amines_Pattern1	369.1131
6PPD-Q TPs	Sulfation of secondary alcohol	379.1322
6PPD-Q TPs	Sulfation of secondary alcohol	381.1478
6PPD-Q TPs	Sulfation of secondary alcohol	383.1635

6PPD-Q TPs	Sulfation of secondary alcohol	385.1791
6PPD-Q TPs	1,3-Dicarbonyl C-glucuronidation	392.0976
6PPD-Q TPs	Sulfation of primary alcohol	393.1115
6PPD-Q TPs	Alkyl-OH-glucuronidation	393.1292
6PPD-Q TPs	Sulfation of primary alcohol	395.1271
6PPD-Q TPs	Sulfation of primary alcohol	397.1428
6PPD-Q TPs	1,3-Dicarbonyl C-glucuronidation	400.1602
6PPD-Q TPs	N-Glucuronidation of hydroxylamine	407.1085
6PPD-Q TPs	Sulfation of primary alcohol	411.122
6PPD-Q TPs	Alkyl-OH-glucuronidation	475.2075
6PPD-Q TPs	Alkyl-OH-glucuronidation	477.2231
6PPD-Q TPs	Alkyl-OH-glucuronidation	479.2388
6PPD-Q TPs	Alkyl-OH-glucuronidation	481.2544
6PPD-Q TPs	Alkyl-OH-glucuronidation	489.1867
6PPD-Q TPs	Alkyl-OH-glucuronidation	491.2024
6PPD-Q TPs	Alkyl-OH-glucuronidation	493.218
6PPD-Q TPs	Alkyl-OH-glucuronidation	507.1973
6PPD-Q TPs	GSH-conjugation of alpha,beta-unsaturated carbonyl	520.1496
6PPD-Q TPs	GSH-conjugation of quinone	522.1653
6PPD-Q TPs	GSH-conjugation of alpha,beta-unsaturated carbonyl	536.1445
6PPD-Q TPs	GSH-conjugation of quinone	538.1602
6PPD-Q TPs	GSH-conjugation of alpha,beta-unsaturated carbonyl	600.2122

6PPD-Q TPs	GSH-conjugation of alpha,beta-unsaturated carbonyl	602.2279
6PPD-Q TPs	GSH-conjugation of alpha,beta-unsaturated carbonyl	604.2435
6PPD-Q TPs	GSH-conjugation of quinone	606.2592
6PPD-Q TPs	GSH-conjugation of alpha,beta-unsaturated carbonyl	618.2228
6PPD-Q TPs	GSH-conjugation of alpha,beta-unsaturated carbonyl	620.2384
6PPD-Q TPs	GSH-conjugation of quinone	622.2541
6PPD-Q TPs	GSH-conjugation of alpha,beta-unsaturated carbonyl	636.2334
6PPD-Q TPs	GSH-conjugation of quinone	638.249

Table S4. Predicted ecotoxicological data of 6PPD, 6PPD-Q and their biotransformation products (based on the US EPA ECOSAR 2.2 software).[15]

Compounds	Fish $LC_{50,\ 96hr}$ ($mg\cdot L^{-1}$)	Daphnid $LC_{50,\ 48hr}$ ($mg\cdot L^{-1}$)	Green algae $EC_{50,\ 96hr}$ ($mg\cdot L^{-1}$)	Earthworm $LC_{50,\ 14d}$ ($mg\cdot L^{-1}$)
6PPD	8.64E-01	6.28E-01	1.30E+00	2.46E+02
6DTP186	1.22E+01	6.50E+00	1.57E+01	2.89E+02
6DTP511	6.06E+01	3.27E+01	6.13E+01	9.31E+02
6DTP431	2.58E+02	1.48E+02	1.17E+02	7.22E+02
6DTP388	3.68E+03	3.88E+02	4.08E+02	8.67E+03
6DTP348	3.40E+03	1.73E+03	8.16E+02	8.04E+02
6DTP428	7.73E+03	3.82E+03	1.61E+03	1.06E+03
6PPD-Q	4.16E-02	8.87E-02	5.53E-02	3.22E+02
6QTP297	4.18E-02	8.88E-02	5.56E-02	3.24E+02
6QTP313	4.42E-02	9.13E-02	6.01E-02	3.49E+02
6QTP315	6.60E-02	1.88E-01	7.05E-02	4.14E+02
6QTP329A	9.04E-02	3.20E-01	8.19E-02	4.84E+02
6QTP331	1.05E-01	3.74E-01	9.44E-02	5.57E+02
6QTP349	1.18E-01	4.30E-01	1.05E-01	6.19E+02
6QTP493	1.96E-01	1.17E+00	1.20E-01	7.21E+02
6QTP477	1.85E-01	9.00E-01	1.32E-01	7.85E+02
6QTP395	3.44E-01	2.34E+00	1.91E-01	1.15E+03
6QTP411	4.81E-01	4.06E+00	2.27E-01	1.37E+03
6QTP317	9.26E+00	2.61E+01	8.03E-01	6.42E+02
6QTP361	1.27E+01	3.74E+01	1.22E+00	7.59E+02
6QTP329	8.20E+00	1.01E+00	1.54E+00	6.20E+02
6QTP345	1.20E+02	3.48E+00	1.36E+01	1.22E+03

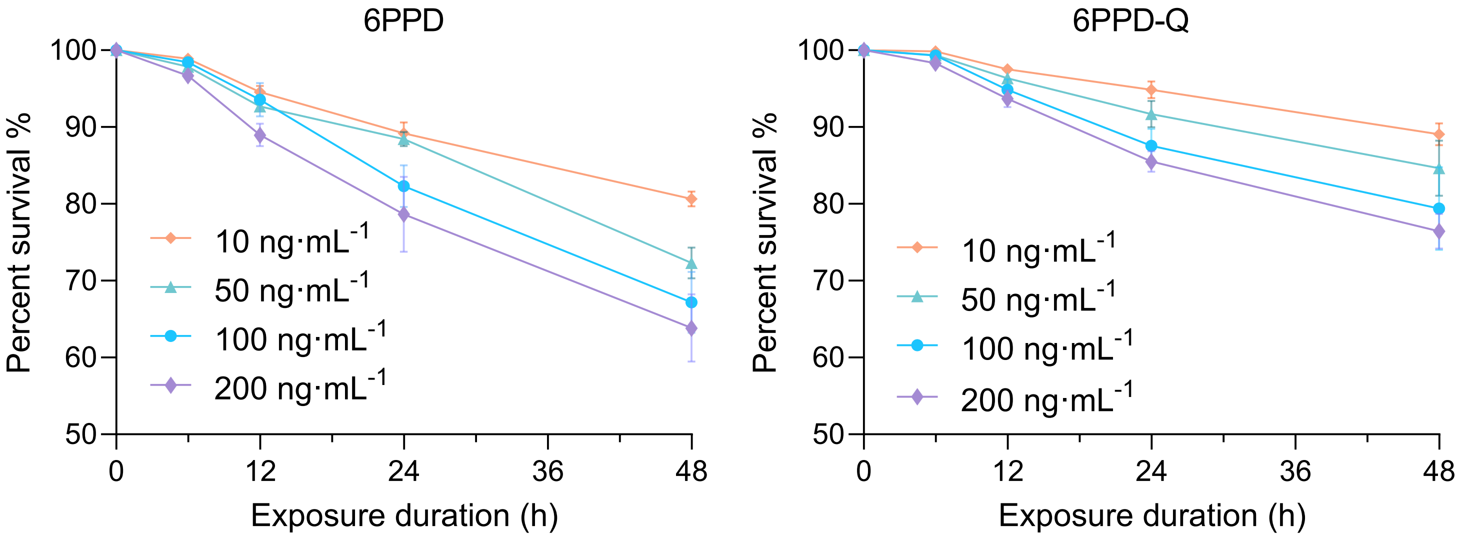

Figure S1. Statistics of lethality of *C. elegans* during exposure to 6PPD and 6PPD-Q with different concentrations. The L4 stage worms were exposed to both contaminants for 48 h in liquid medium. Lethality was counted at 0, 6, 12, 24, and 48h, respectively. No OP_{50} colonies were added into exposure medium to avoid potential metabolism of 6PPD and 6PPD-Q by bacteria. The lethality assays were repeated for three replicates. 24 h and 48 h were chosen as the maximum exposure period for 6PPD and 6PPD-Q, respectively, to avoid quantitative errors due to variations in the number of nematodes (deviation <20%).

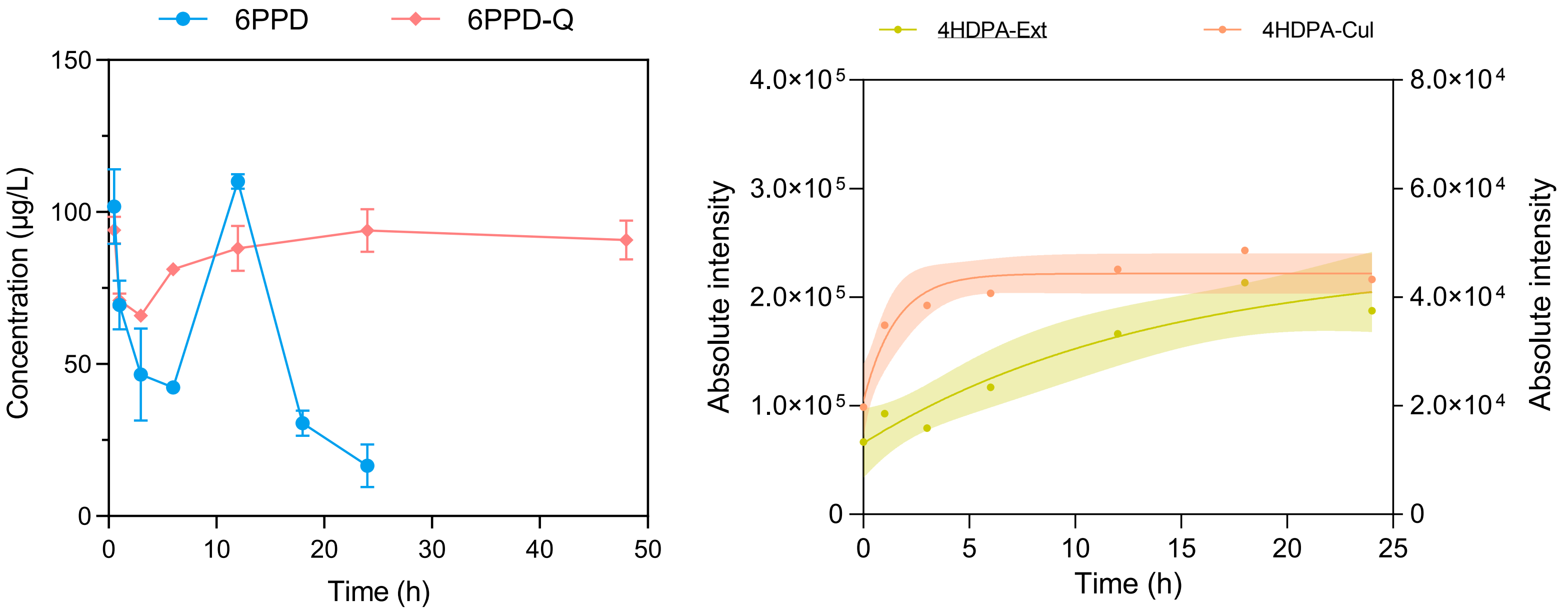

Figure S2. External concentration of 6PPD and 6PPD-Q in the culture (A), and the internal (*C. elegans* extract) and external (culture) abundance of 4-HDPA (B). One time replenishment for 6PPD exposure at 12 h was performed, considering short half-life of 6PPD. Plotted values are the averages of the measured concentrations with curve fitting using lognormal nonlinear regressions. The colored shadow surrounding the regression plot represents the 95% confidence interval. Three biological replicates were performed at each time interval.

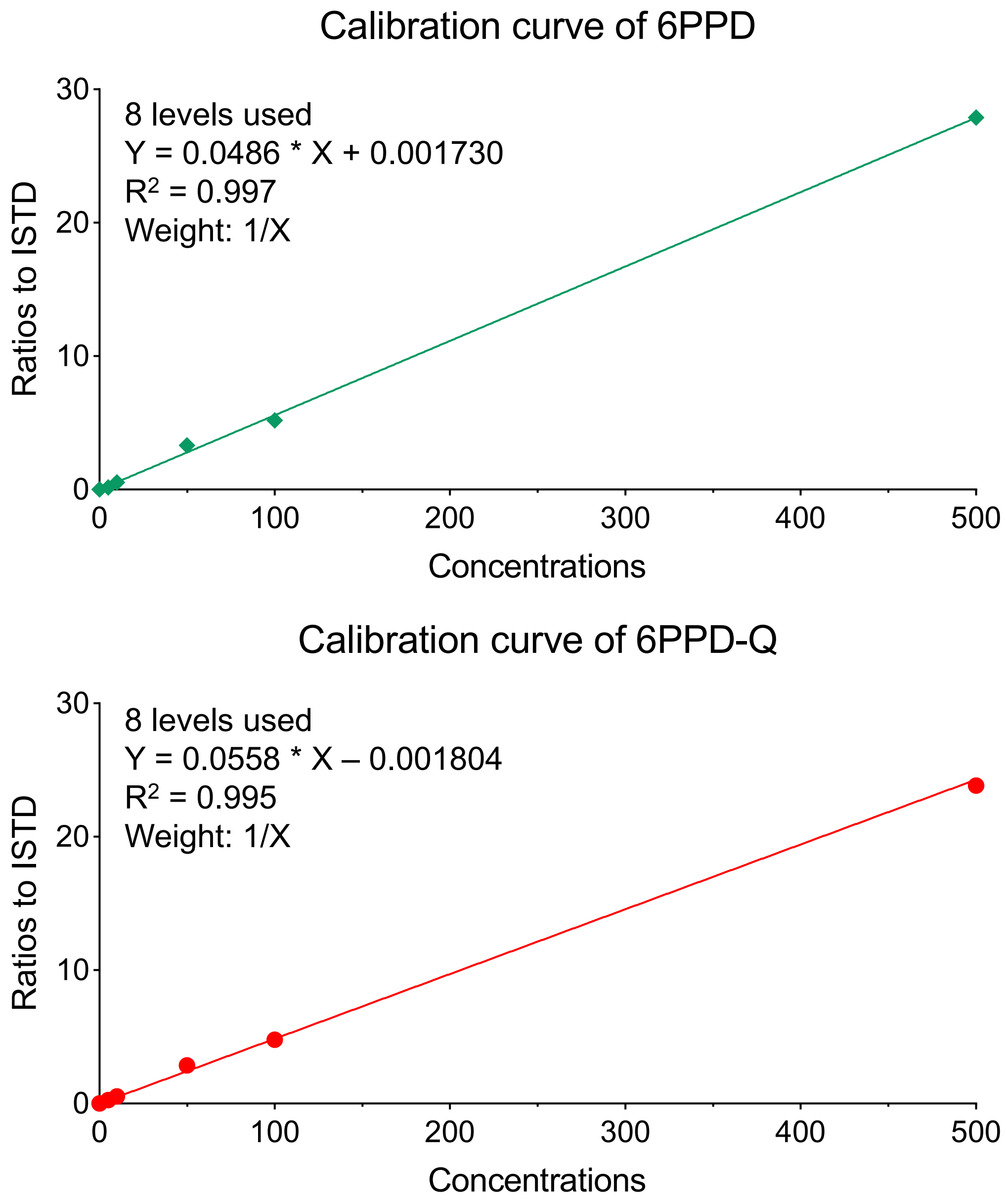

Figure S3. Calibration curves used for the determination of 6PPD and 6PPD-Q.

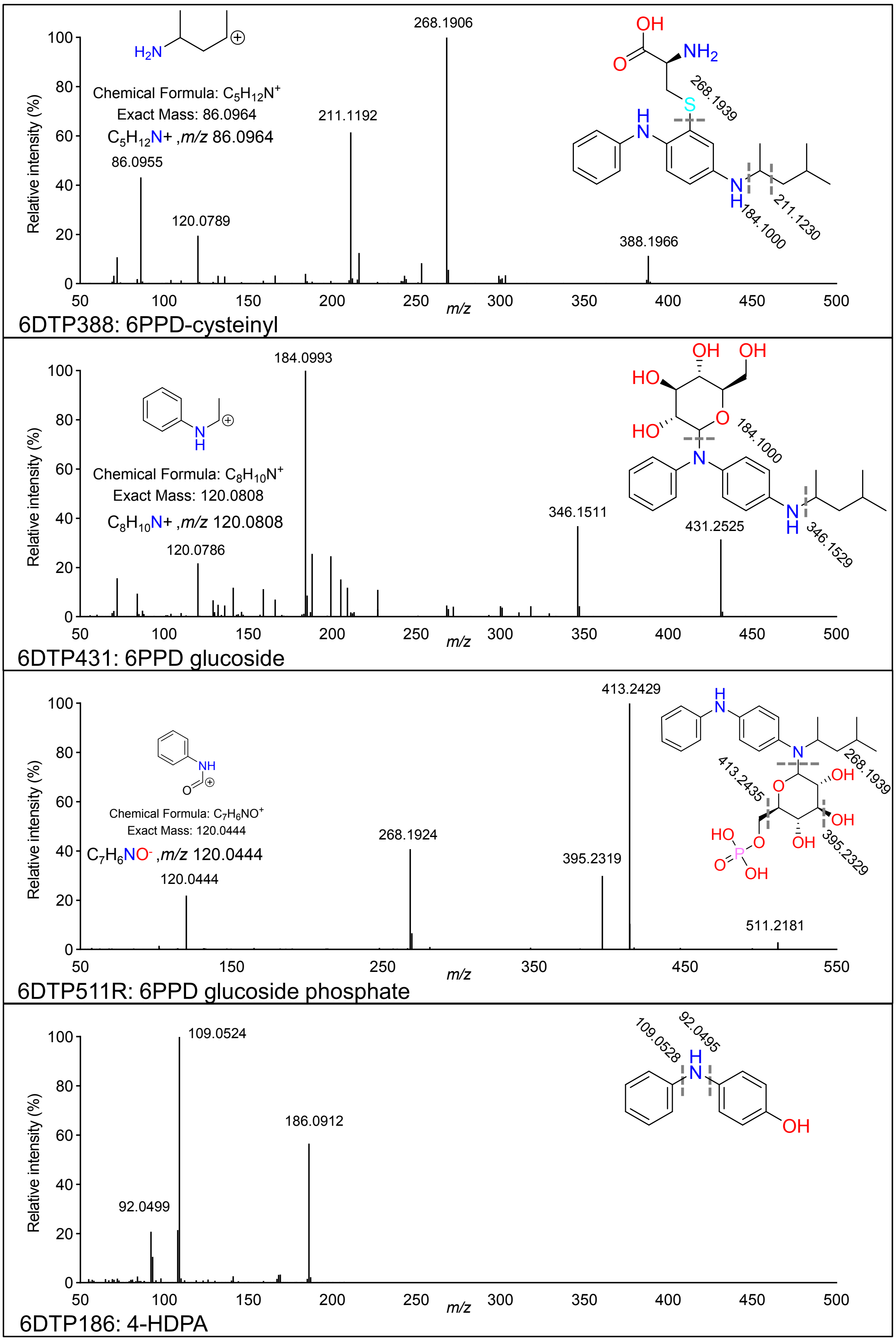

Figure S4. MS/MS fragments of 6DTP388, 6DTP431, 6DTP511R, and 6DTP186.

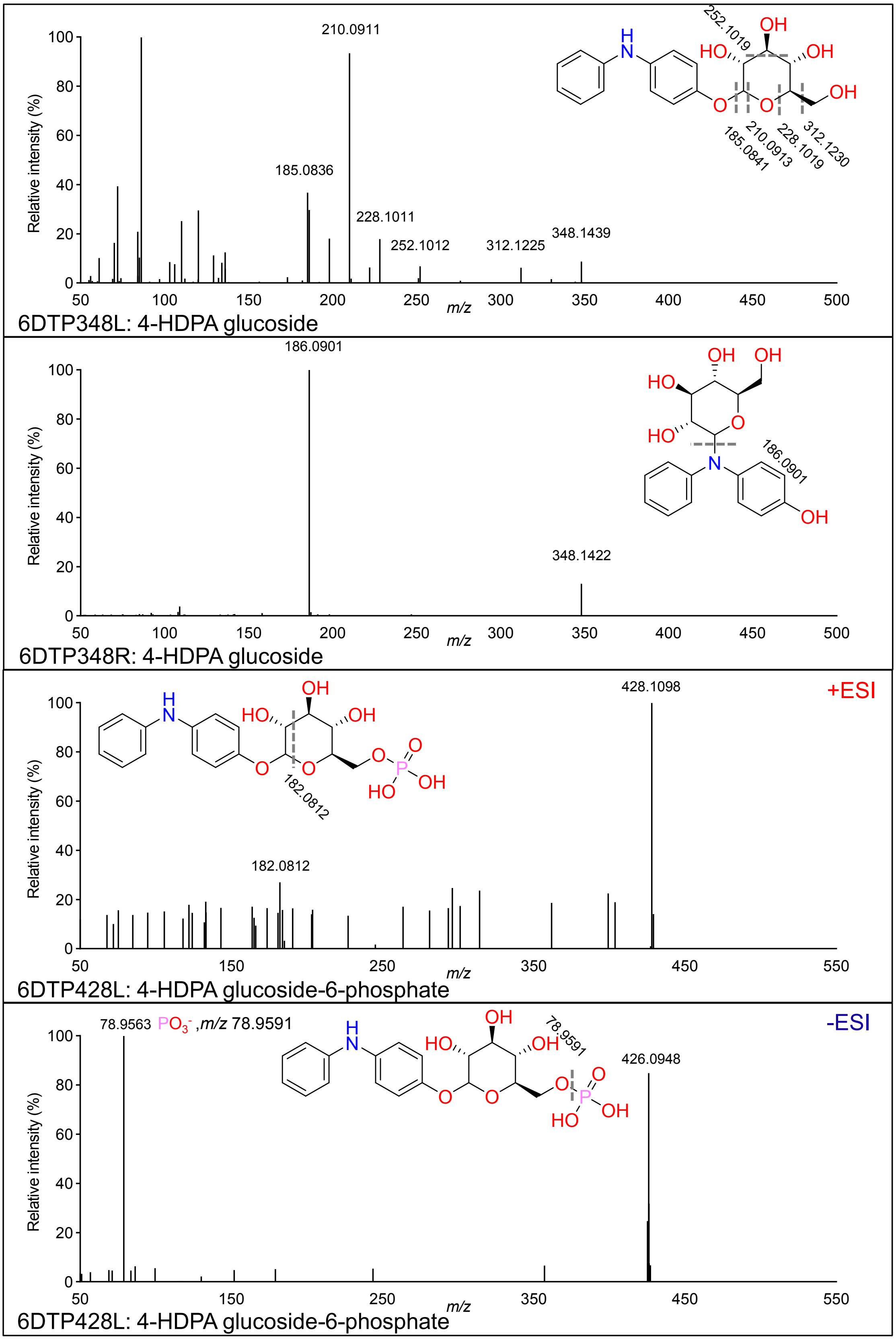

Figure S5. MS/MS fragments of 6DTP348L, 6DTP348R, 6DTP428L (+ESI) and 6DTP428L (-ESI).

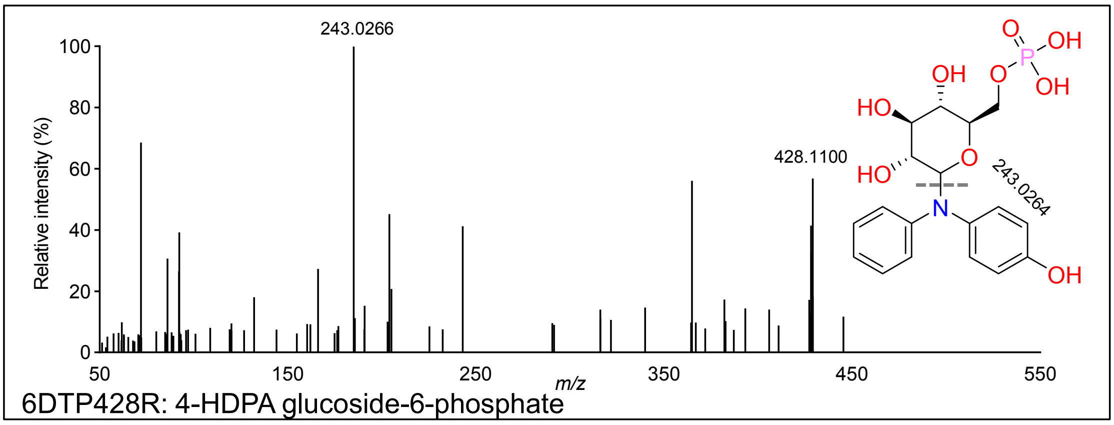

Figure S6. MS/MS fragments of 6DTP428R.

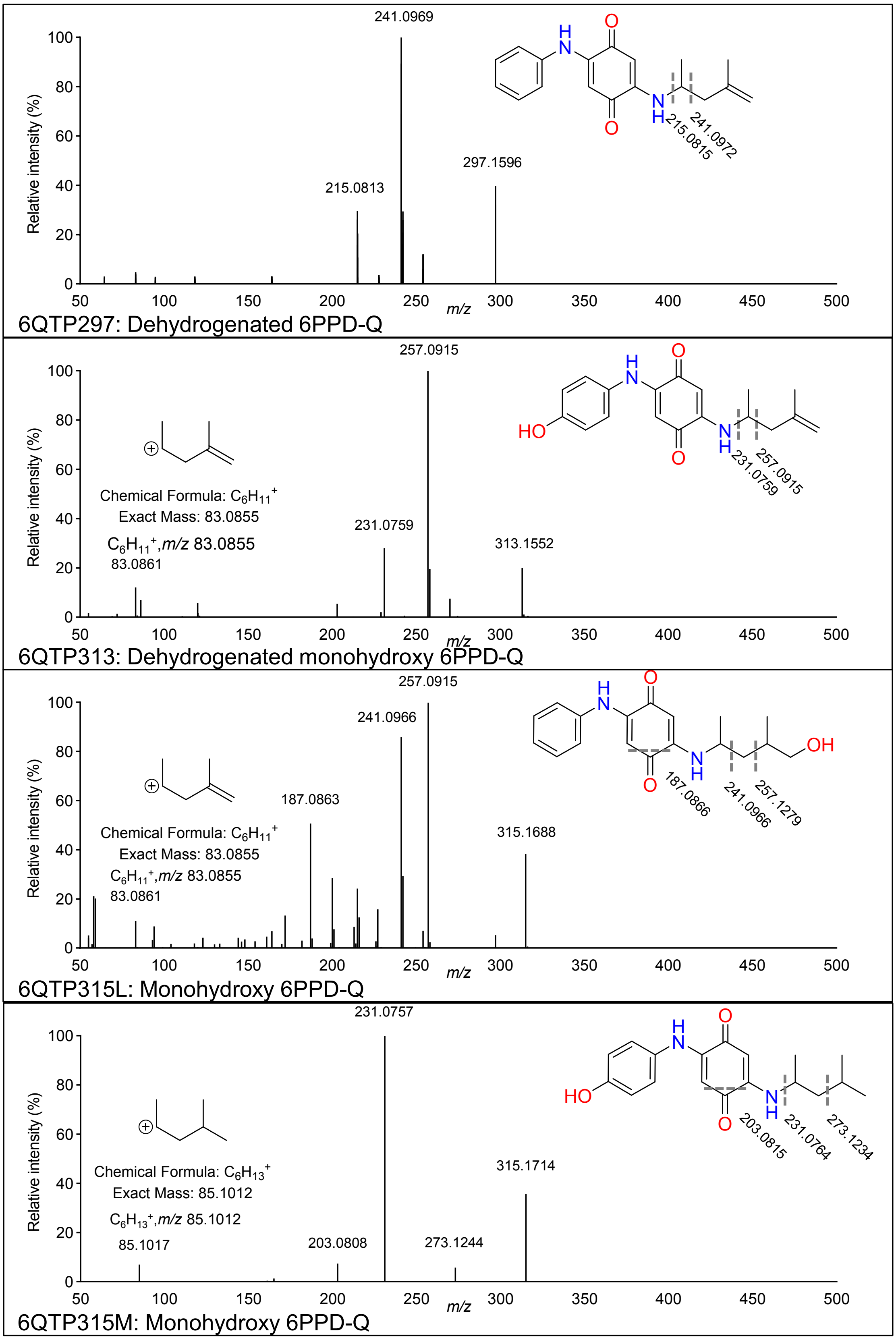

Figure S7. MS/MS fragments of 6QTP297, 6QTP313, 6QTP315L and 6QTP315M. Note that structures of 6QTP297 and 6QTP313 are representative patterns due to the uncertainty of the hydrogenation positions.

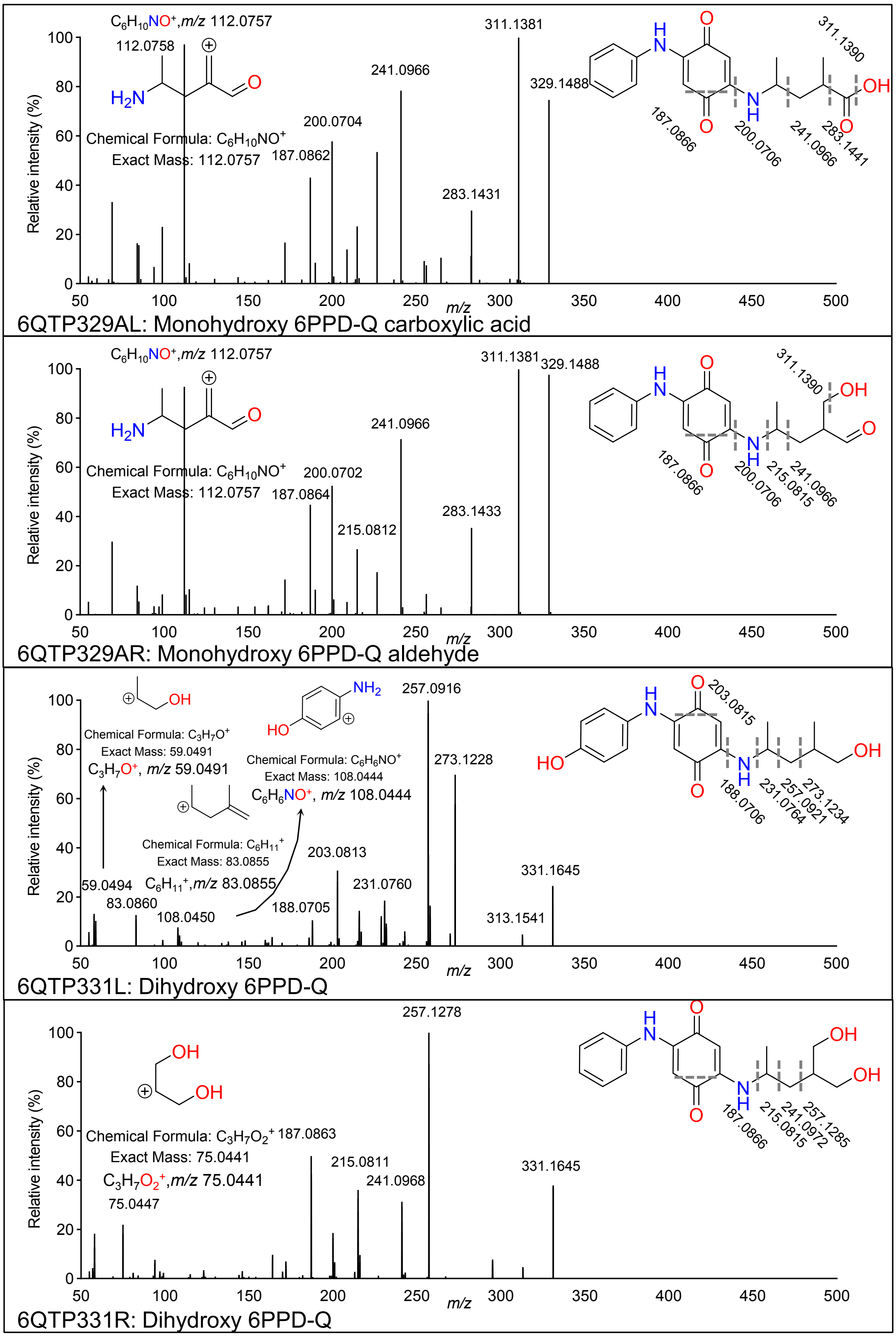

Figure S8. MS/MS fragments of 6QTP329AL, 6QTP329AR, 6QTP331L, and 6QTP331R.

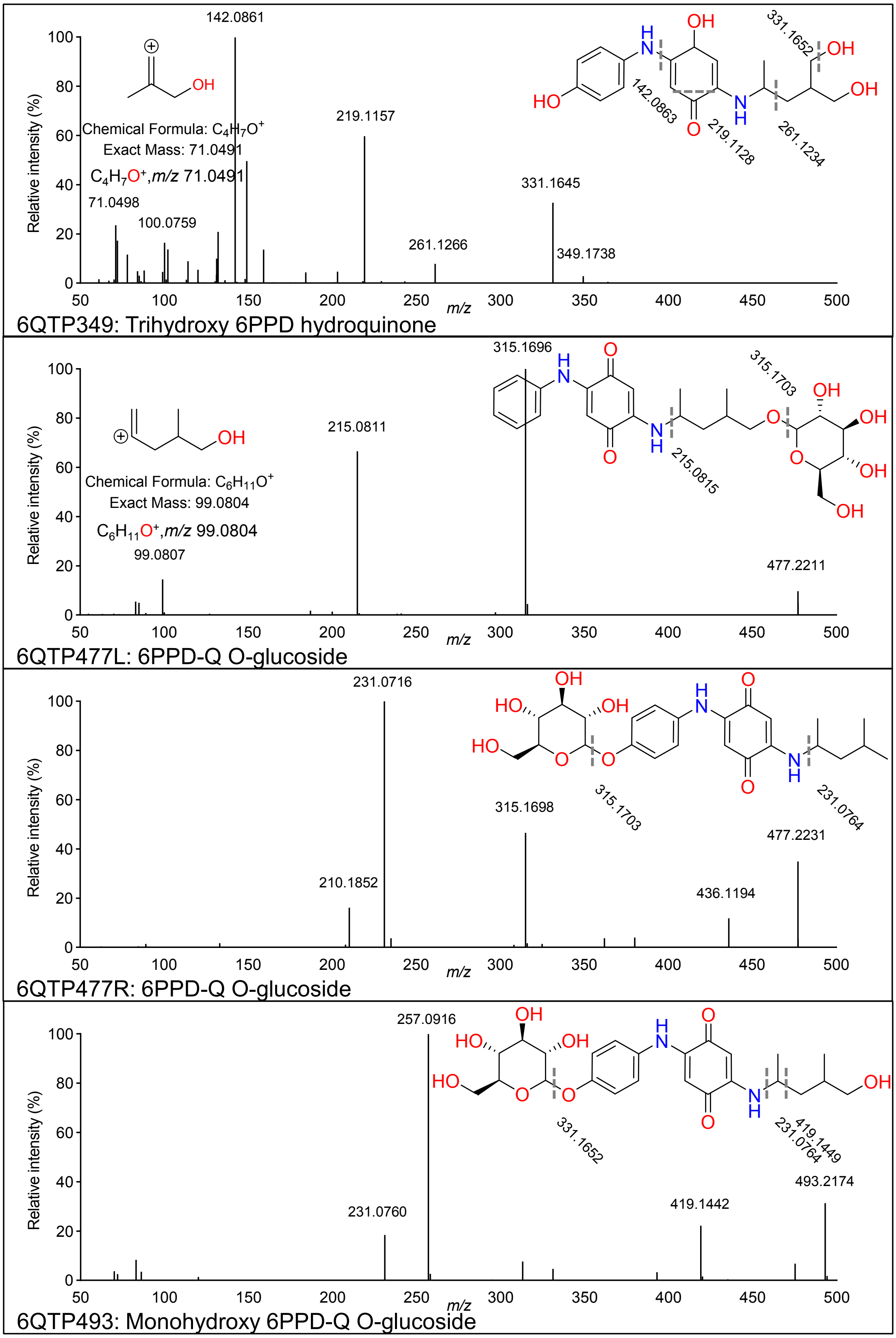

Figure S9. MS/MS fragments of 6QTP349, 6QTP477L, 6QTP477R, and 6QTP493.

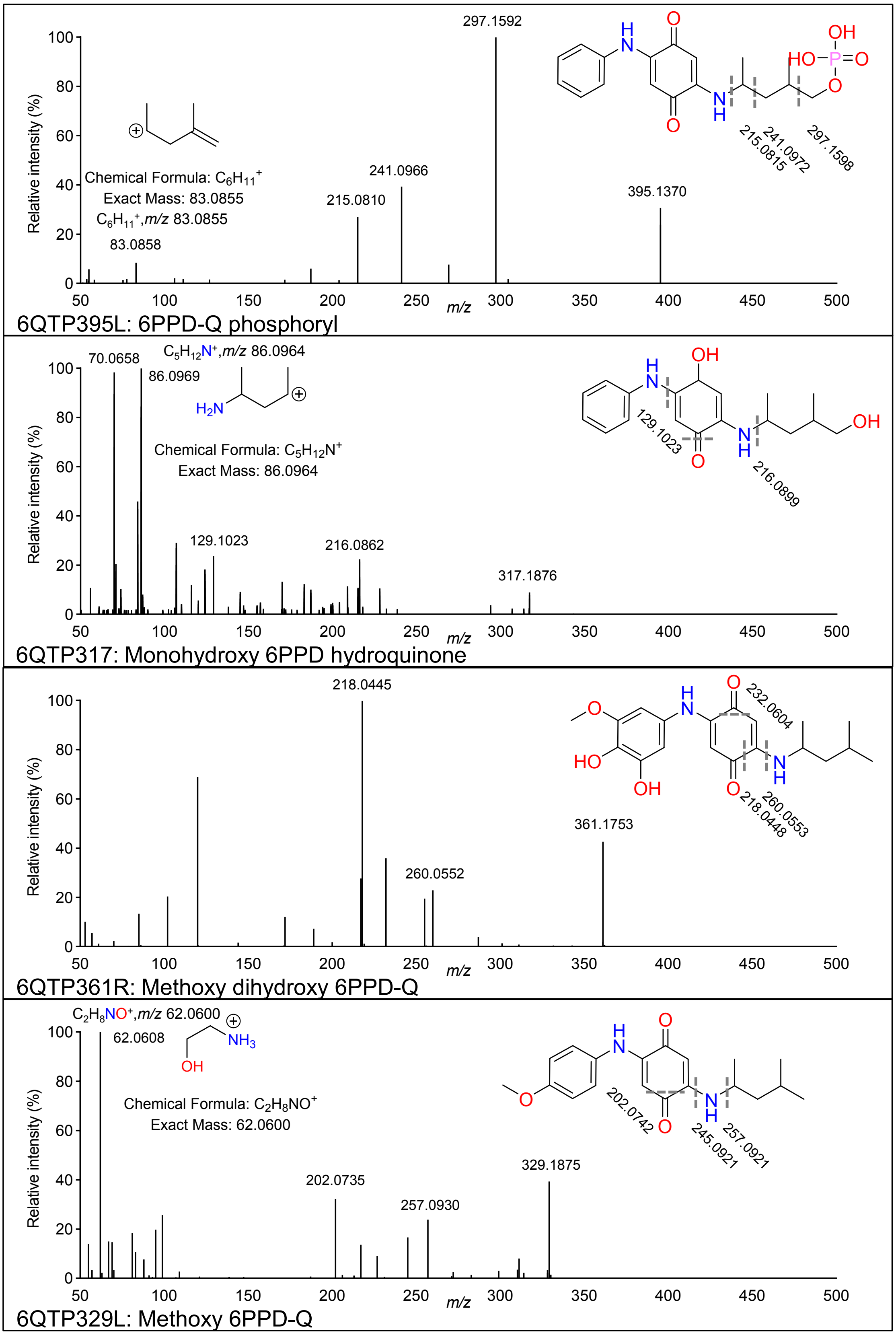

Figure S10. MS/MS fragments of 6QTP395L, 6QTP317, 6QTP361R, and 6QTP329L.

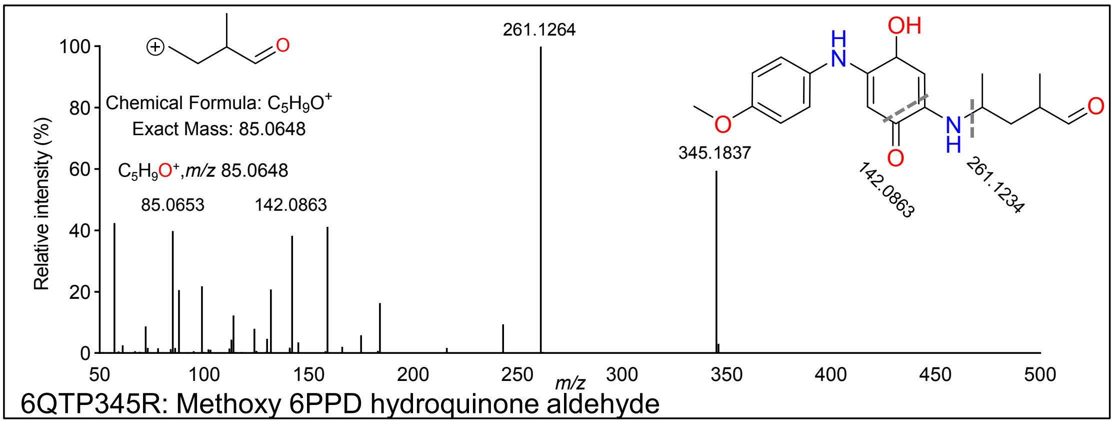

Figure S11. MS/MS fragments of 6QTP345R.

References

1. Epstein, H. F.; Shakes, D. C., *Caenorhibditus elegans: modern biological analysis of an organism*. Academic Press: 1995; Vol. 48.

2. Ristau, K.; Akgül, Y.; Bartel, A. S.; Fremming, J.; Müller, M. T.; Reiher, L.; Stapela, F.; Splett, J. P.; Spann, N., Toxicity in relation to mode of action for the nematode Caenorhabditis elegans: Acute‐to‐chronic ratios and quantitative structure‐activity relationships. *Environ. Toxicol. Chem.* **2015,** *34*, (10), 2347-2353.

3. Höss, S.; Sanders, D.; van Egmond, R., Determining the toxicity of organic compounds to the nematode Caenorhabditis elegans based on aqueous concentrations. *Environ. Sci. Pollut. Res.* **2023,** *30*, (42), 96290-96300.

4. Chowdhury, M. I.; Sana, T.; Panneerselvan, L.; Sivaram, A. K.; Megharaj, M., Perfluorooctane sulfonate (PFOS) induces several behavioural defects in Caenorhabditis elegans that can also be transferred to the next generations. *Chemosphere* **2022,** *291*, 132896.

5. Nair, P.; Sun, J.; Xie, L.; Kennedy, L.; Kozakiewicz, D.; Kleywegt, S. M.; Hao, C.; Byun, H.; Barrett, H.; Baker, J.; Monaghan, J.; Krogh, E. T.; Song, D.; Peng, H., Synthesis and Toxicity Evaluation of p-Phenylenediamine-Quinones. *Environ Sci Technol* **2025,** *59*, (15), 7485-7494.

6. Zhang, Y. Y.; Huang, J. W.; Liu, Y. H.; Zhang, J. N.; Huang, Z.; Liu, Y. S.; Zhao, J. L.; Ying, G. G., In vitro metabolism of the emerging contaminant 6PPD-quinone in human and rat liver microsomes: Kinetics, pathways, and mechanism. *Environ Pollut* **2024,** *345*, 123514.

7. Ankley, P. J.; da Silva, F. C.; Montgomery, D.; Schultz, M.; Ed, S. K.; Hecker, M.; Brinkmann, M., Biotransformation of 6PPD-quinone In Vitro Using RTL-W1 Cell Line. *Environ. Sci. Technol. Lett.* **2024,** *11*, (7), 687-693.

8. Halama, J. J.; McKane, R. B.; Barnhart, B. L.; Pettus, P. P.; Brookes, A. F.; Adams, A. K.; Gockel, C. K.; Djang, K. S.; Phan, V.; Chokshi, S. M.; Graham, J. J.; Tian, Z.; Peter, K. T.; Kolodziej, E. P., Watershed analysis of urban stormwater contaminant 6PPD-Quinone hotspots and stream concentrations using a process-based ecohydrological model. *Front Environ Sci* **2024,** *12*, 1-12.

9. Hiki, K.; Asahina, K.; Kato, K.; Yamagishi, T.; Omagari, R.; Iwasaki, Y.; Watanabe, H.; Yamamoto, H., Acute Toxicity of a Tire Rubber-Derived Chemical, 6PPD Quinone, to Freshwater Fish and Crustacean Species. *Environ. Sci. Technol. Lett.* **2021,** *8*, (9), 779-784.

10. Grasse, N.; Seiwert, B.; Massei, R.; Scholz, S.; Fu, Q.; Reemtsma, T., Uptake and Biotransformation of the Tire Rubber-derived Contaminants 6-PPD and 6-PPD Quinone in the Zebrafish Embryo (Danio rerio). *Environ Sci Technol* **2023,** *57*, (41), 15598-15607.

11. Zhang, J.; Cao, G.; Wang, W.; Qiao, H.; Chen, Y.; Wang, X.; Wang, F.; Liu, W.; Cai, Z., Stable isotope-assisted mass spectrometry reveals in vivo distribution, metabolism, and excretion of tire rubber-derived 6PPD-quinone in mice. *Sci. Total Environ.* **2024,** *912*, 169291.

12. Castan, S.; Sherman, A.; Peng, R.; Zumstein, M. T.; Wanek, W.; Huffer, T.; Hofmann, T., Uptake, Metabolism, and Accumulation of Tire Wear Particle-Derived Compounds in Lettuce. *Environ Sci Technol* **2023,** *57*, (1), 168-178.

13. Gao, J.; Ellis, L. B. M.; Wackett, L. P., The University of Minnesota Biocatalysis/Biodegradation Database: improving public access. *Nucleic Acids Res.* **2009,** *38*, (suppl_1), D488-D491.

14. Wishart, D. S.; Tian, S.; Allen, D.; Oler, E.; Peters, H.; Lui, Vicki W.; Gautam, V.; Djoumbou-Feunang, Y.; Greiner, R.; Metz, Thomas O., BioTransformer 3.0—a web server for accurately predicting metabolic transformation products. *Nucleic Acids Res.* **2022,** *50*, (W1), W115-W123.

15. USEPA, ECOSAR. Available at: https://www.epa.gov/tsca-screening-tools/ecological-structure-activity-relationships-ecosar-predictive-model (*accessed* October 11, 2022)
